# Supplementary material for: Micro-costing from healthcare professional’s perspective and acceptability of cutaneous leishmaniasis diagnostic tools in Morocco: A mixed-methods study
Source: PLOS Glob Public Health. 2024 Mar 28;4(3):e0002534. doi: 10.1371/journal.pgph.0002534 (PMC10977798; doi:10.1371/journal.pgph.0002534)
Supplement: S1 Video — (DOCX) [file pgph.0002534.s001.docx]

**S1_Video. CL RDT video demonstration**

**[
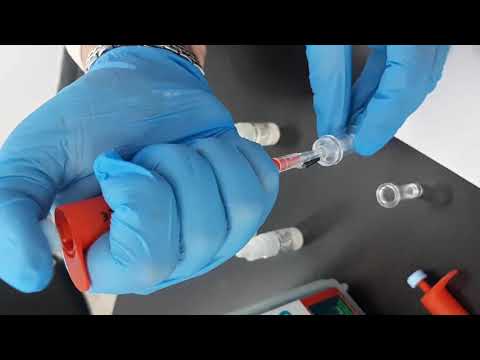
](https://www.youtube.com/embed/MAfVfQD41x8?feature=oembed)**

**Video link:** [**https://www.youtube.com/embed/MAfVfQD41x8**](https://www.youtube.com/embed/MAfVfQD41x8)
