# Supplementary material for: Micro-costing from healthcare professional’s perspective and acceptability of cutaneous leishmaniasis diagnostic tools in Morocco: A mixed-methods study
Source: PLOS Glob Public Health. 2024 Mar 28;4(3):e0002534. doi: 10.1371/journal.pgph.0002534 (PMC10977798; doi:10.1371/journal.pgph.0002534)
Supplement: S1 Fig — (DOCX) [file pgph.0002534.s002.docx]

**S1_Fig. Zoonotic and Anthroponotic cutaneous leishmaniasis cases from 1998 until 2020 in Morocco**

Bennis I. Cutaneous leishmaniasis in Morocco: psychosocial burden and simplified diagnosis. [Belgium]: Antwerp; 2018; completed by the Moroccan Ministry of Health information

ZCL: Zoonotic cutaneous leishmaniasis; ACL: Anthroponotic cutaneous leishmaniasis
