# Supplementary material for: Micro-costing from healthcare professional’s perspective and acceptability of cutaneous leishmaniasis diagnostic tools in Morocco: A mixed-methods study
Source: PLOS Glob Public Health. 2024 Mar 28;4(3):e0002534. doi: 10.1371/journal.pgph.0002534 (PMC10977798; doi:10.1371/journal.pgph.0002534)
Supplement: S1 Text — (DOCX) [file pgph.0002534.s003.docx]

**S1_Text. Topic guide interview for health professionals**

**01 -** Tell me, how is the first visit of a suspicious patient with a skin leishmaniasis lesion (CL)? (Different scenarios depending on the patient’s cases and the health professional experience?

**02 -** Is the care pathway the same for both men and women?

**03 -** Is the care pathway the same for children compared to older people?

**04 -** And for the case of multiple lesions, what do you do?

**05 -** Did you hear about a rapid diagnostic test for cutaneous leishmaniasis?

**Now, I'll show you a short video demonstrating how the CL rapid diagnostic test is used.**

**After watching the video, please continue the interview with the following questions:**

**06 -** What is the amount of workload needed to do RDT compared to microscopy?

**07 -** What do you expect from the use of this CL RDT compared to microscopy?

**08 -** What do you think about your patient's feelings concerning both diagnostic tools?

**09 -** What is the general description of the patient diagnostic pathway?

**10 -** What is the diagnostic confirmation percentage of the suspected CL patients?

**11 -** What is the time between diagnostic demand and diagnostic confirmation?

**12 -** What is your recommendation to enhance CL diagnostic in primary health centres?

**13 -** If the RDT would show a similar or better accuracy than microscopy, would you agree to recommend its generalisation in all endemic rural primary health centres?
