# Supplementary material for: Micro-costing from healthcare professional’s perspective and acceptability of cutaneous leishmaniasis diagnostic tools in Morocco: A mixed-methods study
Source: PLOS Glob Public Health. 2024 Mar 28;4(3):e0002534. doi: 10.1371/journal.pgph.0002534 (PMC10977798; doi:10.1371/journal.pgph.0002534)
Supplement: S2 Text — (DOCX) [file pgph.0002534.s004.docx]

**S2_Text. Thematic guide for the in-depth personal interview**

1) Would healthcare professionals be more willing to use rapid diagnostic tests or microscopy to diagnose cutaneous leishmaniasis? And why do you think so?

2) What is the accepted and tolerable length of time from your point of view between the request for diagnosis and the confirmation of the diagnosis?

3) Do you think the result will be more accurate in confirming cutaneous leishmaniasis using this rapid test or using microscopy?

4) If the rapid test shows similar accuracy to microscopy, which of the two diagnostic tools do you prefer to use as a first line?

And if, for example, the rapid test performed first remains negative or inconclusive, would you agree to do microscopy as a second choice or is it enough for you to keep the initial result of the rapid test?

5) If you are the health decision-maker, what can you suggest as a diagnostic tool for cutaneous leishmaniasis, and at what cost would you agree to buy it from the manufacturer or sell it to patients?

6) What is the actual pathway that the patient should follow to get a CL diagnostic result?

7) In the case of CL RDT availability, what would be the patient’s diagnostic pathway?
