# Supplementary material for: Micro-costing from healthcare professional’s perspective and acceptability of cutaneous leishmaniasis diagnostic tools in Morocco: A mixed-methods study
Source: PLOS Glob Public Health. 2024 Mar 28;4(3):e0002534. doi: 10.1371/journal.pgph.0002534 (PMC10977798; doi:10.1371/journal.pgph.0002534)
Supplement: S3 Text — (DOCX) [file pgph.0002534.s005.docx]

**S3_Text. Questionnaire of laboratory and primary health centres professionals about CL diagnostic (RDT, microscopy)**

**The province names**

**Name of health facility**

**Date of doing the questionnaire**

**1. Diagnostic test type used: (For participants doing both tests previously, please tick just CL RDT as a response)**

🞐 Microscopy

🞐 CL Detect Rapid ™

**2.1 The average time required to complete diagnostic test (Hours and minutes):**

**2.2 The minimum time required to complete diagnostic test (Hours and minutes):**

**2.3 The maximum time required to complete a diagnostic test (Hours and minutes):**

**3. How many health professionals (HP) are involved in performing the diagnostic test?**

**What are their respective functions? What is the monthly salary and average time spent** **on this test?**

- Each HP involved (1 = nurse; 2 =doctor; 3 = tech lab; 4 = other)
- Monthly salary in dirhams (MAD) for each HP involved
- Average time spent on the test (Hours and Minutes) for each HP involved

**4. What type of material was issued for this diagnostic test? Please notice the name and the quantity (for treating one lesion or how many ones)**

**5. What is the average annual cost associated with this diagnostic method?**

**(For example, costs such as microscopy maintenance, control** of the **quality** of the **microscope** **laboratory, etc.)**

**Details: .....................................................**

**6. How many suspected cases of** CL have been managed annually **in this health facility (estimation)?**

**7. How many microscopy tests does this health facility carry out or request (approximately) per year to diagnose cutaneous leishmaniasis and other diseases?**
