# Supplementary material for: Micro-costing from healthcare professional’s perspective and acceptability of cutaneous leishmaniasis diagnostic tools in Morocco: A mixed-methods study
Source: PLOS Glob Public Health. 2024 Mar 28;4(3):e0002534. doi: 10.1371/journal.pgph.0002534 (PMC10977798; doi:10.1371/journal.pgph.0002534)
Supplement: S1 Table — (DOCX) [file pgph.0002534.s006.docx]

**S1_Table. Cutaneous leishmaniasis 2019’s epidemiological data and yearly CL lesions in the study area**

| Province | PHC in the study | Nb of CL patients in 2019 in the selected PHC | <18 years old | ≥18 years old | Gender M/F for ≥ 18 yo | The overall percentage of lesions per patient 18 yo and more (*) | | | | Weighted average lesions** | Yearly 2019 number of ZCL patients in these provinces | Yearly 2019 number of ACL patients in these provinces |
| --- | --- | --- | --- | --- | --- | --- | --- | --- | --- | --- | --- | --- |
|  |  |  |  |  |  | One lesion | Two lesions | 3 to 4 | Five and more |  |  |  |
| Errachidia | 12 | 182 | 70 | 112 | 56/56 | 44% | 24% | 28% | 4% | 2,2 | 381 | 0 |
| Ouarzazate | 7 | 630 | 304 | 326 | 162/164 | 46% | 29% | 17% | 8% | 2,2 | 638 | 107 |
| Sefrou | 4 | 33 | 23 | 10 | 04/06 | 43% | 28% | 21% | 8% | 2,2 | 0 | 35 |
| Tinghir | 12 | 285 | 208 | 77 | 28/49 | 65% | 24% | 9% | 2% | 1,7 | 481 | 402 |
| Total in *L.major* areas | 19 | 812 | 374 | 438 | 218/220 | 45% | 27% | 22% | 6% | 2,2 | 1500 |  |
| Total in *L.tropica* areas | 16 | 318 | 231 | 87 | 32/55 | 54% | 26% | 15% | 5% | 2,0 |  | 544 |

(*) Information is found from the previous study done in 2017 in the same provinces (Ref) and completed by the Moroccan Ministry of Health information.

(**)For Errachidia the weighted average lesions = ((44×1.1)+(24×2.2)+(28×3.6)+(4×5))/100 = 2,2

​1.1 is allocated for one lesion, 2.2 for two lesions, 3.6 for three to four lesions, and 5 for five or more lesions.

Errachidia and Ouarzazate are considered L major or zoonotic ZCL areas, and Sefrou and Tinghir are considered *L. tropica* or anthropotic ACL areas.

Ref (Bennis & al 2018) Bennis I, Verdonck K, El Khalfaoui N, Riyad M, Fellah H, Dujardin JC, et al. Accuracy of a Rapid Diagnostic Test Based on Antigen Detection for the Diagnosis of Cutaneous Leishmaniasis in Patients with Suggestive Skin Lesions in Morocco. The American Journal of Tropical Medicine and Hygiene. 2018 Sep 5;99(3):716–22.
