# Supplementary material for: Micro-costing from healthcare professional’s perspective and acceptability of cutaneous leishmaniasis diagnostic tools in Morocco: A mixed-methods study
Source: PLOS Glob Public Health. 2024 Mar 28;4(3):e0002534. doi: 10.1371/journal.pgph.0002534 (PMC10977798; doi:10.1371/journal.pgph.0002534)
Supplement: S2 Table — (DOCX) [file pgph.0002534.s007.docx]

**S2_Table. List of anonymised participants, their localisation, profile, and previous knowledge about CL RDT**

| **Initial in-depth interviews** | **Province** | **Profile** | **Sex** | **Health type facility** | **Health facility name** | **Use of CL RDT** |
| --- | --- | --- | --- | --- | --- | --- |
| ID01 | Z | Nurse | M | PHC CSC | Sekoura*** | **Used** |
| ID02 | Z | Nurse | F | Laboratory* | Ouarzazate town | Known |
| ID03 | S | Nurse | M | PHC CSU | Menzel | Not Known |
| ID04 | S | Med | F | PHC CSC | Azzaba | Not Known |
| ID05 | S | Nurse | M | PHC CSC | Ribat El Kheir | Not Known |
| ID06 | S | Nurse | M | PHC CSU | Chahd Baha Sef town*** | **Used** |
| ID07 | S | Nurse | M | Laboratory* | Sefrou Town | Known |
| ID08 | Z | Nurse | M | PHC CSC | Tarmight*** | **Used** |
| ID09 | Z | Med | M | PHC CSC | Ghessate | Known |
| ID10 | Z | Nurse | F | PHC CSC | Ait Kadif | Not Known |
| ID11 | Z | Nurse | M | PHC CSC | Tassoumate | Not Known |
| ID12 | Z | Med | M | PHC CSC | Sidi Daoud | Not Known |
| ID13 | Z | Nurse | M | PHC CSU | Douar Chams | Not Known |
| ID15 | E | Med | F | PHC CSU | Tinejdad | Not Known |
| ID16 | E | Nurse | F | PHC CSU | Erfoud | Not Known |
| ID17 | E | Nurse | M | PHC CSU | Goulmima | Known |
| ID18 | E | Nurse | M | PHC CSU | Boutalamine Errachidia | Not Known |
| ID19 | E | Nurse | F | PHC CSC | Aoufous | Not Known |
| ID20 | E | Nurse | F | PHC CSC | Sifa | Not Known |
| ID21 | E | Nurse | M | PHC CSC | Mellaab Hennabou | Not Known |
| ID22 | E | Tech | F | PHC CSU | Jorf | Not Known |
| ID23 | E | Nurse | M | PHC CSC | Tadighoust | Not Known |
| ID24 | E | Nurse | F | PHC CSU | My Ail Chrif | Not Known |
| ID25 | E | Med | M | PHC CSU | Abderahman Sah Errachidia | Known |
| ID26 | E | Nurse | M | PHC CSC | Ferkla*** | **Used** |
| ID27 | E | Tech | M | Laboratory* | Errachidia Town | Known |
| ID28 | T | Nurse | M | PHC CSU | Kelaa Megouna A | Not Known |
| ID29 | T | Nurse | F | PHC CSC | Ait Tazarine | Known |
| ID30 | T | Nurse | F | PHC CSC | Tassouite | Not Known |
| ID31 | T | Nurse | M | PHC CSC | Sedrae Jbel Soufli | Known |
| ID32 | T | Nurse | F | PHC DR | Bouteghrar | Known |
| ID33 | T | Nurse | M | PHC DR | Ait Hemd | Known |
| ID34 | T | Nurse | M | PHC CSC | Kmiss Dades | Known |
| ID35 | T | Nurse | F | PHC DR | Sarghine | Not Known |
| ID36 | T | Nurse | F | PHC CSU | Boumalen Dades A | Known |
| ID37 | T | Nurse | M | PHC CSC | Ait Ouassif*** | **Used** |
| ID38 | T | Nurse | F | PHC CSC | Ait Hammo Osaid | Not Known |
| ID39 | T | Nurse | F | PHC CSC | Ait Youl | Known |
| ID40 | T | Nurse | M | Laboratory* | Boumalen Dades B | Known |
| ID41 | T | Tech | M | Laboratory* | Kelaa Megouna B*** | **Used** |
| ID14** | Z | Med | F | PHC CSU | Douar Chams | Known |
| ID42** | E | Nurse | F | PHC CSC | Sifa | Not Known |
| ID43** | E | Nurse | F | PHC CSC | Aoufous | Not Known |
| ID44** | E | Nurse | M | PHC CSU | Erfoud | **Used** |
| ID45** | E | Nurse | F | PHC CSU | My Ail Chrif | Not Known |
| ID46** | Z | Nurse | M | PHC CSC | Sekoura | Known |

(*) 5 laboratories supporting the primary health facilities level (in addition to the 35 PHC)

(**) ID 14, 42, 43, 44, 45 and 46 are the participants in the final 06 focused In-depth interviews

(***) The six distinct healthcare facilities were healthcare professionals participating in the study, had prior hands-on experience with the CL-RDT

E: Errachidia province

S: Sefrou province

T: Tinghir province

Z: Ouarzazate province

Med: Medical doctor

Tech: Technician laboratory

PHC CSU: Primary health centre with medical activities in an urban area

PHC CSC: Primary health centre with medical activities in a rural area

PHC DR: Primary health centre with only nursing activities in a rural area
