# Supplementary material for: Micro-costing from healthcare professional’s perspective and acceptability of cutaneous leishmaniasis diagnostic tools in Morocco: A mixed-methods study
Source: PLOS Glob Public Health. 2024 Mar 28;4(3):e0002534. doi: 10.1371/journal.pgph.0002534 (PMC10977798; doi:10.1371/journal.pgph.0002534)
Supplement: S3 Table — (DOCX) [file pgph.0002534.s008.docx]

**S3_Table. The unitary equipment and laboratory material costs for both CL microscopy and RDT expressed in MAD (1 USD = 9,6 MAD)**

| **Item** | **For both procedures** | | **For Microscopy confirmation, only** | | **For CL RDT confirmation, only** | |
| --- | --- | --- | --- | --- | --- | --- |
|  | **Quantity needed for one lesion** | **Unitary costs in MAD** | **The amount required for one lesion** | **Unitary costs in MAD** | **The amount required for one lesion** | **Unitary costs in MAD** |
| **For scraping cutaneous leishmaniasis samples** | | | | | | |
| Gloves | One pair | 3,00 [1,5-6,0] |  | |  | |
| Cotton | Small piece | 0.02 [0,01-0,04] |  |  |  |  |
| Physiological serum | 5 to 10 cc | 1,00 [0,5-3,0] |  |  |  |  |
| Local anaesthetic (*Emla*©) | 1 mg | 3,00 [1,0-4,0] |  |  |  |  |
| Band-aid | One piece | 0,50 [0,2-2,0] |  |  |  |  |
| Sterile Blood Lancet or surgical cutter | 1 unit | 0,30 [0,2-1,5] |  |  |  |  |
| Povidone-iodine | 3 to 5 cc | 0.03 [0,01-0,4] |  |  |  |  |
| Ointment antibiotic (Chlortetracycline 3%) | 1 mg | 1,00 [0,5-2,0] |  |  |  |  |
| Small plaster | One piece | 0,50 [0,2-1,0] |  |  |  |  |
| Band-aid or big Plaster | One piece | 1,00 [0,5-2,0] |  |  |  |  |
| **For doing the technique of CL RDT** (buffers dental broach, reaction tube, pipette tips)** | | | | | | |
| Strip test with buffers (InBios™ cost) |  | |  | | 1 unit | 62 [46-102] |
| Dental broach |  |  |  |  | 1 unit | 2,0 [1- 4] |
| Reaction cup |  |  |  |  | 1 cup | 0.45 [ 0,2-1,1] |
| Pipette tip |  |  |  |  | One tip | 0.2 [0,1-0,6] |
| Eppendorf tube |  |  |  |  | 1 unit | 0.4 [0,3-1,4] |
| **For doing smear sample colouration and microscopic lecture** | | | | | | |
| Gloves |  | | One pair | 3,00 [1,5-6,0] |  | |
| Glass slides |  |  | 2 to 4 | 1,00 [0,5 – 2,0] |  |  |
| Slides fixation in ethanol (60 ml reused 30 times) |  |  | 2ml for each slide | 0,06 [0,05-0,10] |  |  |
| Slides in bleu Giemsa colouration (20 ml reused 100 times) |  |  | 0.2ml for an emerged slot of 4 slides | 0,08 [0,05-1,15] |  |  |
| Laboratory purified Water (80 ml reused 100 times) |  | | 0.8ml for an emerged slot of 4 slides | 0.01 [0,01-0.03] |  | |
|  | | | | | | |
| Subtotal costs |  | 10,35 [4,6-21,9] |  | 5,15 [2,1-9,3] |  | 65,05 [47,6-109,1] |
| **Total unitary cost for Mic vs CL RDT (in MAD)** | | | **15,5 [6,7-31,2]** | | **75,4 [52,2-131]** | |

(*) For Microscopy, the cup used for emersion, or the slides supports and organizer slides box, the drop of oil for lecture emersion in the light microscope, and the microscope 100x magnification purchase or yearly maintenance, the paper to notice the results are not included in the costs.

(**) CL RDT has different costs depending on the quantity, updated version, and manufacturing costs. The unit means the cost is 62 MAD [46-102] when the purchase is conditioned as a package of 25 strips (including the buffer lysis, buffer chase, positive control, and negative control). The additional equipment for performing the RDT correctly needs a dental broach, pipette tip and reaction tube. The 25µl pipette and the notebook to notice the results are not included in the costs.

MAD=Moroccan Dirham. November 2019 rate change (1 USD=9.6 MAD).
