# Supplementary material for: Micro-costing from healthcare professional’s perspective and acceptability of cutaneous leishmaniasis diagnostic tools in Morocco: A mixed-methods study
Source: PLOS Glob Public Health. 2024 Mar 28;4(3):e0002534. doi: 10.1371/journal.pgph.0002534 (PMC10977798; doi:10.1371/journal.pgph.0002534)
Supplement: S6 Table — (DOCX) [file pgph.0002534.s011.docx]

**S6_Table. Extraction of the full qualitative tree codes by NVivo software analysis**

| Name | Files | References | Used for this paper |
| --- | --- | --- | --- |
| 00 CL First visits | **1** | **1** |  |
| 001 Before visiting the PHC | **21** | **27** | **No** |
| 002 PHC visits | **42** | **71** | **No** |
| 01 Participants Characteristics | **41** | **78** |  |
| 011 HP experience with CL | **3** | **3** | **Yes** |
| New HP | **13** | **18** | **Yes** |
| Yes Experienced | **34** | **53** | **Yes** |
| 012 Proximity of Provincial laboratory | **1** | **1** | **Yes** |
| Less than 20 Km | **30** | **47** | **Yes** |
| More than 20 Km | **11** | **19** | **Yes** |
| 02 CL statistics | **2** | **2** |  |
| 021 % of CL cases confirmed from suspected | **37** | **47** | **Yes** |
| 022 % standard treatment effectiveness | **11** | **14** | **No** |
| 023 Other statistics | **26** | **43** | **No** |
| 03 Lesions characteristics | **10** | **15** |  |
| 031 one lesion | **10** | **12** | **No** |
| 032 multiple lesions | **26** | **36** | **No** |
| 04 CL patient pathway into Health facility | **5** | **5** |  |
| 041 Regular pathway Following the MoH CL guideline management | **43** | **103** | **Yes** |
| 042 No respect for the protocol pathway | **27** | **47** | **Yes** |
| 05 Diagnostic preferences | **5** | **5** |  |
| 051 Clinical presumption | **9** | **13** | **Yes** |
| Confidence in the clinical presumption | **15** | **25** | **Yes** |
| Difficulties in doing smear confirmation | **15** | **23** | **Yes** |
| 052 Both, it depends on | **22** | **31** | **Yes** |
| 053 Do smear confirmation systematically | **32** | **58** | **Yes** |
| Prepare the smear at the Health Centre | **26** | **37** | **Yes** |
| Sent patients to Laboratory | **38** | **75** | **Yes** |
| 06 Time out (Délai de retard) | **5** | **6** |  |
| 061 PHC time out | **13** | **19** | **Yes** |
| 062 Microscopy results time out | **43** | **127** | **Yes** |
| 063 TTT time out | **29** | **69** | **No** |
| 07 Performed CL Diagnostic & and time | **4** | **4** |  |
| 071 Time to perform Microscopy | **32** | **76** | **Yes** |
| 072 RDT previously performed | **2** | **2** | **Yes** |
| 0721 RDT never known before | **23** | **24** | **Yes** |
| 0722 RDT know but not used | **16** | **17** | **Yes** |
| 0723 RDT known and used | **7** | **10** | **Yes** |
| 073 Time to perform RDT | **31** | **55** | **Yes** |
| 08 Perception of the usefulness of RDT | **9** | **10** |  |
| 81 For patient interest | **45** | **135** | **Yes** |
| 82 For Health professional interest | **43** | **105** | **Yes** |
| 83 Workload due to RDT | **35** | **95** | **Yes** |
| 09 Patients' willingness to pay | **12** | **16** |  |
| 91 Drugs to buy | **27** | **46** | **No** |
| 92 Laboratory costs | **33** | **76** | **Yes** |
| 93 Travel costs | **40** | **79** | **Yes** |
| 94 RDT private cost & and date of production | **52** | **119** | **Yes** |
| 95 Other expenses | **24** | **35** | **No** |
| 10 Recommendations | **9** | **10** |  |
| 100 Future recommended pathways | **8** | **8** | **Yes** |
| 1001 New Diagnostic Pathway | **45** | **156** | **Yes** |
| 1002 New TTT pathway | **32** | **75** | **No** |
| 101 RDT | **48** | **222** | **Yes** |
| 102 Microscopy | **33** | **59** | **Yes** |
| 103 Treatment | **46** | **143** | **No** |
| 104 Others | **39** | **95** | **Yes** |
| 11 Fears & HP Communication effects | **31** | **58** |  |
| 111 Patients fear the disease | **33** | **55** | **No** |
| 1111 CL is not a deadly disease | **19** | **22** | **No** |
| 1112 Visibility of the CL on the body | **19** | **24** | **No** |
| 1113 Impact of Scars | **22** | **39** | **No** |
| 112 Other ideas | **29** | **63** | **No** |
