# Supplementary material for: Micro-costing from healthcare professional’s perspective and acceptability of cutaneous leishmaniasis diagnostic tools in Morocco: A mixed-methods study
Source: PLOS Glob Public Health. 2024 Mar 28;4(3):e0002534. doi: 10.1371/journal.pgph.0002534 (PMC10977798; doi:10.1371/journal.pgph.0002534)

| **!**pip install pandas |
| --- |

# In [115…

Requirement already satisfied: pandas in c:\users\admin\appdata\local\programs\pytho n\python311\lib\site-packages (1.5.3)

Requirement already satisfied: python-dateutil>=2.8.1 in c:\users\admin\appdata\loca l\programs\python\python311\lib\site-packages (from pandas) (2.8.2)

Requirement already satisfied: pytz>=2020.1 in c:\users\admin\appdata\local\programs

\python\python311\lib\site-packages (from pandas) (2022.7.1)

Requirement already satisfied: numpy>=1.21.0 in c:\users\admin\appdata\local\program s\python\python311\lib\site-packages (from pandas) (1.24.2)

Requirement already satisfied: six>=1.5 in c:\users\admin\appdata\local\programs\pyt hon\python311\lib\site-packages (from python-dateutil>=2.8.1->pandas) (1.16.0)

| **import** pandas **as** pd |
| --- |

# In [116…

| **!**pip install openpyxl |
| --- |

# In [117…

Requirement already satisfied: openpyxl in c:\users\admin\appdata\local\programs\pyt hon\python311\lib\site-packages (3.1.2)

Requirement already satisfied: et-xmlfile in c:\users\admin\appdata\local\programs\p ython\python311\lib\site-packages (from openpyxl) (1.1.0)

| df **=** pd**.**read_excel(r'E:\0 Projects\2- EMRO TDR small grant June 2018--2019\0 Manusc |
| --- |

# In [118…

| *#Step 1: Install SALib*  **!**pip install SALib  *#Step 2: Import Required Libraries* **import** numpy **as** np **from** SALib.sample **import** saltelli **from** SALib.analyze **import** sobol  *#Step 3: Define the Problem You will need to define the problem by specifying the n* problem **=** {  'num_vars': 9,  'names': ['Previous Knowledge of CL RDT', 'Diagnostic tool manipulated', 'HP wo 'bounds': [  [0, 1], *# previous_knowledge (binary variable)*  [0, 1], *# diagnostic_tool (Microscopy = yes = 1)*  [20, 62], *# hp_working_time*  [1, 2], *# hp_needed (just one =1)*  [0, 1], *# self_transfer (Sampling in the PHC without patient transfer=1)*  [0, 1], *# driver_transfer (MoH Driver involved=1)*  [14, 432], *# patient_transport_cost*  [0, 1], *# main_lab_tech_involved (binary variable)*  [0, 1], *# admin_moh_cost*  *# Other bounds for remaining variables*  ]  }  *#You'll need to adjust the bounds to match the specific ranges for each variable in* |
| --- |

# In [119…

Requirement already satisfied: SALib in c:\users\admin\appdata\local\programs\python

\python311\lib\site-packages (1.4.7)

Requirement already satisfied: matplotlib>=3.2.2 in c:\users\admin\appdata\local\pro grams\python\python311\lib\site-packages (from SALib) (3.7.1)

Requirement already satisfied: multiprocess in c:\users\admin\appdata\local\programs

\python\python311\lib\site-packages (from SALib) (0.70.14)

Requirement already satisfied: numpy>=1.20.3 in c:\users\admin\appdata\local\program s\python\python311\lib\site-packages (from SALib) (1.24.2)

Requirement already satisfied: pandas>=1.1.2 in c:\users\admin\appdata\local\program s\python\python311\lib\site-packages (from SALib) (1.5.3)

Requirement already satisfied: scipy>=1.7.3 in c:\users\admin\appdata\local\programs

\python\python311\lib\site-packages (from SALib) (1.11.1)

Requirement already satisfied: contourpy>=1.0.1 in c:\users\admin\appdata\local\prog rams\python\python311\lib\site-packages (from matplotlib>=3.2.2->SALib) (1.1.0)

Requirement already satisfied: cycler>=0.10 in c:\users\admin\appdata\local\programs

\python\python311\lib\site-packages (from matplotlib>=3.2.2->SALib) (0.11.0)

Requirement already satisfied: fonttools>=4.22.0 in c:\users\admin\appdata\local\pro grams\python\python311\lib\site-packages (from matplotlib>=3.2.2->SALib) (4.40.0)

Requirement already satisfied: kiwisolver>=1.0.1 in c:\users\admin\appdata\local\pro grams\python\python311\lib\site-packages (from matplotlib>=3.2.2->SALib) (1.4.4)

Requirement already satisfied: packaging>=20.0 in c:\users\admin\appdata\local\progr ams\python\python311\lib\site-packages (from matplotlib>=3.2.2->SALib) (23.1)

Requirement already satisfied: pillow>=6.2.0 in c:\users\admin\appdata\local\program s\python\python311\lib\site-packages (from matplotlib>=3.2.2->SALib) (10.0.0)

Requirement already satisfied: pyparsing>=2.3.1 in c:\users\admin\appdata\local\prog rams\python\python311\lib\site-packages (from matplotlib>=3.2.2->SALib) (3.1.0)

Requirement already satisfied: python-dateutil>=2.7 in c:\users\admin\appdata\local

\programs\python\python311\lib\site-packages (from matplotlib>=3.2.2->SALib) (2.8.2)

Requirement already satisfied: pytz>=2020.1 in c:\users\admin\appdata\local\programs

\python\python311\lib\site-packages (from pandas>=1.1.2->SALib) (2022.7.1)

Requirement already satisfied: dill>=0.3.6 in c:\users\admin\appdata\local\programs

\python\python311\lib\site-packages (from multiprocess->SALib) (0.3.6)

Requirement already satisfied: six>=1.5 in c:\users\admin\appdata\local\programs\pyt hon\python311\lib\site-packages (from python-dateutil>=2.7->matplotlib>=3.2.2->SALi b) (1.16.0)

| **def** evaluate_model(params):  previous_knowledge, diagnostic_tool, hp_working_time, hp_needed, self_transfer, output **=** (  52.3   - 35.8 ***** diagnostic_tool   **+** 7.4 ***** previous_knowledge  **+** 0.4 ***** hp_working_time   - 3.9 ***** hp_needed   **+** 29.6 ***** main_lab_tech_involved   - 2.4 ***** self_transfer **+** 7.1 ***** driver_transfer   **+** 0.007 ***** patient_transport_cost  **+** 1.0 ***** admin_moh_cost  )  **return** output  *# IS 45 MAD UNITARY CL Detect RDT STRIP >> 83 MAD THRESHOLD* |
| --- |

# In [120…

| **from** SALib.sample.sobol **import** sample **as** sobol_sample **from** SALib.analyze.sobol **import** analyze **as** sobol_analyze | | |
| --- | --- | --- |
|  | |  |
| param_values **=** sobol_sample(problem, | | N**=**352) *# N is the number of samples* |
|  | |  |
| Y **=** | np**.**array([evaluate_model(params) | **for** params **in** param_values]) |
|  |  |  |
| Si **=** | sobol**.**analyze(problem, Y) |  |
|  |  |  |
| S1 **=** | Si['S1'] |  |
| ST **=** | Si['ST'] |  |
|  |  |  |
| print("First order sensitivity indices:", Si['S1']) | | |

# In [121…

In [135…

In [136…

In [137…

In [138…

# In [139…

First order sensitivity indices: [0.01883915 0.49538869 0.10695015 0.00541889 0.0026

9011 0.0239119

0.00405201 0.33220764 0.0012769 ]

| print("Total sensitivity indices:", Si['ST']) |
| --- |

# In [140…

Total sensitivity indices: [2.08701656e-02 4.99567796e-01 1.06213541e-01 5.78187863e

-03

2.19504345e-03 1.91841328e-02 3.24910639e-03 3.32978141e-01

3.82862011e-04]

| **import** matplotlib.pyplot **as** plt  *# Assuming Si is the object containing the sensitivity indices* S1_values **=** Si['S1']  *# Using names from the problem definition* names **=** problem['names']  plt**.**bar(names, S1_values) plt**.**ylabel('First-order Sensitivity Index (S1)') plt**.**xticks(rotation**=**90) plt**.**show() |
| --- |

# In [141…


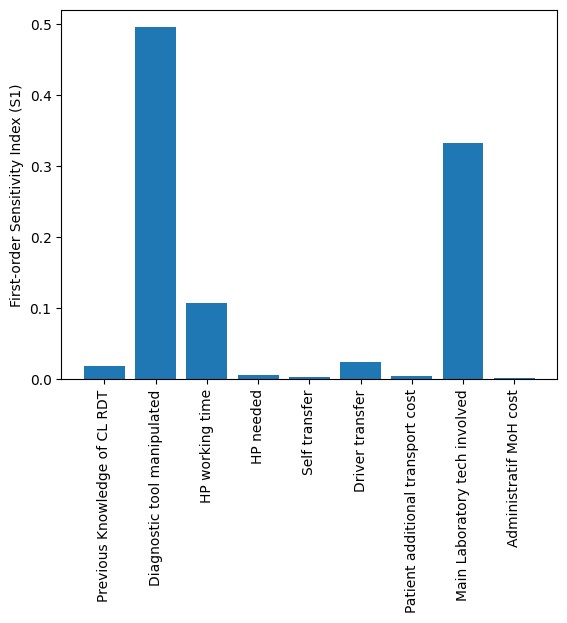


| **import** matplotlib.pyplot **as** plt  *# Assuming Si is the object containing the sensitivity indices* ST_values **=** Si['ST']  *# Using names from the problem definition* names **=** problem['names']  plt**.**bar(names, ST_values) plt**.**ylabel('Total sensitivity indices (ST)') plt**.**xticks(rotation**=**90) plt**.**show() |
| --- |

# In [142…


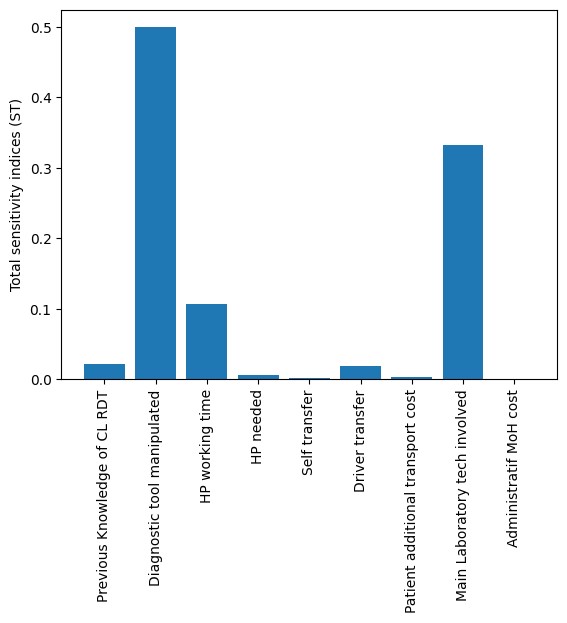


| *#Scenario 1*  *# Define your specific values for the parameters* previous_knowledge **=** 1 hp_working_time **=** 30 hp_needed **=** 1 main_lab_tech_involved **=** 0 self_transfer **=** 0 driver_transfer **=** 0 patient_transport_cost **=** 0 admin_moh_cost **=** 0 microscopy_cost **=** 115 *# Fixed cost for Microscopy*  *# Define the function for calculating the RDT cost*  **def** calculate_rdt_cost(previous_knowledge, hp_working_time, hp_needed, main_lab_tec diagnostic_tool **=** 1 *# Representing CL RDT*  output **=** ( |
| --- |

# In [143…

| 52.3   - 35.8 ***** diagnostic_tool   **+** 7.4 ***** previous_knowledge  **+** 0.4 ***** hp_working_time   - 3.9 ***** hp_needed   **+** 29.6 ***** main_lab_tech_involved   - 2.4 ***** self_transfer **+** 7.1 ***** driver_transfer   **+** 0.007 ***** patient_transport_cost  **+** 1.0 ***** admin_moh_cost  )  **return** output  *# Iterate through different unitary prices for CL RDT*  **for** rdt_price **in** range(5, 115): *# Assuming a range from 35 to 114*  rdt_cost **=** calculate_rdt_cost(previous_knowledge, hp_working_time, hp_needed, m **if** rdt_cost **<** microscopy_cost:  print(f"At a unitary price of {rdt_price} MAD, CL RDT is advantageous.") **else**:  print(f"At a unitary price of {rdt_price} MAD, CL RDT is not advantageous." |
| --- |

At a unitary price of 5 MAD, CL RDT is advantageous.

At a unitary price of 6 MAD, CL RDT is advantageous.

At a unitary price of 7 MAD, CL RDT is advantageous.

At a unitary price of 8 MAD, CL RDT is advantageous.

At a unitary price of 9 MAD, CL RDT is advantageous.

At a unitary price of 10 MAD, CL RDT is advantageous.

At a unitary price of 11 MAD, CL RDT is advantageous.

At a unitary price of 12 MAD, CL RDT is advantageous.

At a unitary price of 13 MAD, CL RDT is advantageous.

At a unitary price of 14 MAD, CL RDT is advantageous.

At a unitary price of 15 MAD, CL RDT is advantageous.

At a unitary price of 16 MAD, CL RDT is advantageous.

At a unitary price of 17 MAD, CL RDT is advantageous.

At a unitary price of 18 MAD, CL RDT is advantageous.

At a unitary price of 19 MAD, CL RDT is advantageous.

At a unitary price of 20 MAD, CL RDT is advantageous.

At a unitary price of 21 MAD, CL RDT is advantageous.

At a unitary price of 22 MAD, CL RDT is advantageous.

At a unitary price of 23 MAD, CL RDT is advantageous.

At a unitary price of 24 MAD, CL RDT is advantageous.

At a unitary price of 25 MAD, CL RDT is advantageous.

At a unitary price of 26 MAD, CL RDT is advantageous.

At a unitary price of 27 MAD, CL RDT is advantageous.

At a unitary price of 28 MAD, CL RDT is advantageous.

At a unitary price of 29 MAD, CL RDT is advantageous.

At a unitary price of 30 MAD, CL RDT is advantageous.

At a unitary price of 31 MAD, CL RDT is advantageous.

At a unitary price of 32 MAD, CL RDT is advantageous.

At a unitary price of 33 MAD, CL RDT is advantageous.

At a unitary price of 34 MAD, CL RDT is advantageous.

At a unitary price of 35 MAD, CL RDT is advantageous.

At a unitary price of 36 MAD, CL RDT is advantageous.

At a unitary price of 37 MAD, CL RDT is advantageous.

At a unitary price of 38 MAD, CL RDT is advantageous.

At a unitary price of 39 MAD, CL RDT is advantageous.

At a unitary price of 40 MAD, CL RDT is advantageous.

At a unitary price of 41 MAD, CL RDT is advantageous.

At a unitary price of 42 MAD, CL RDT is advantageous.

At a unitary price of 43 MAD, CL RDT is advantageous.

At a unitary price of 44 MAD, CL RDT is advantageous.

At a unitary price of 45 MAD, CL RDT is advantageous.

At a unitary price of 46 MAD, CL RDT is advantageous.

At a unitary price of 47 MAD, CL RDT is advantageous.

At a unitary price of 48 MAD, CL RDT is advantageous.

At a unitary price of 49 MAD, CL RDT is advantageous.

At a unitary price of 50 MAD, CL RDT is advantageous.

At a unitary price of 51 MAD, CL RDT is advantageous.

At a unitary price of 52 MAD, CL RDT is advantageous.

At a unitary price of 53 MAD, CL RDT is advantageous.

At a unitary price of 54 MAD, CL RDT is advantageous.

At a unitary price of 55 MAD, CL RDT is advantageous.

At a unitary price of 56 MAD, CL RDT is advantageous.

At a unitary price of 57 MAD, CL RDT is advantageous.

At a unitary price of 58 MAD, CL RDT is advantageous.

At a unitary price of 59 MAD, CL RDT is advantageous.

At a unitary price of 60 MAD, CL RDT is advantageous.

At a unitary price of 61 MAD, CL RDT is advantageous.

At a unitary price of 62 MAD, CL RDT is advantageous.

At a unitary price of 63 MAD, CL RDT is advantageous.

At a unitary price of 64 MAD, CL RDT is advantageous.

At a unitary price of 65 MAD, CL RDT is advantageous.

At a unitary price of 66 MAD, CL RDT is advantageous.

At a unitary price of 67 MAD, CL RDT is advantageous.

At a unitary price of 68 MAD, CL RDT is advantageous.

At a unitary price of 69 MAD, CL RDT is advantageous.

At a unitary price of 70 MAD, CL RDT is advantageous.

At a unitary price of 71 MAD, CL RDT is advantageous.

At a unitary price of 72 MAD, CL RDT is advantageous.

At a unitary price of 73 MAD, CL RDT is advantageous.

At a unitary price of 74 MAD, CL RDT is advantageous.

At a unitary price of 75 MAD, CL RDT is advantageous.

At a unitary price of 76 MAD, CL RDT is advantageous.

At a unitary price of 77 MAD, CL RDT is advantageous.

At a unitary price of 78 MAD, CL RDT is advantageous.

At a unitary price of 79 MAD, CL RDT is advantageous.

At a unitary price of 80 MAD, CL RDT is advantageous.

At a unitary price of 81 MAD, CL RDT is advantageous.

At a unitary price of 82 MAD, CL RDT is advantageous.

At a unitary price of 83 MAD, CL RDT is not advantageous.

At a unitary price of 84 MAD, CL RDT is not advantageous.

At a unitary price of 85 MAD, CL RDT is not advantageous.

At a unitary price of 86 MAD, CL RDT is not advantageous.

At a unitary price of 87 MAD, CL RDT is not advantageous.

At a unitary price of 88 MAD, CL RDT is not advantageous.

At a unitary price of 89 MAD, CL RDT is not advantageous.

At a unitary price of 90 MAD, CL RDT is not advantageous.

At a unitary price of 91 MAD, CL RDT is not advantageous.

At a unitary price of 92 MAD, CL RDT is not advantageous.

At a unitary price of 93 MAD, CL RDT is not advantageous.

At a unitary price of 94 MAD, CL RDT is not advantageous.

At a unitary price of 95 MAD, CL RDT is not advantageous.

At a unitary price of 96 MAD, CL RDT is not advantageous.

At a unitary price of 97 MAD, CL RDT is not advantageous.

At a unitary price of 98 MAD, CL RDT is not advantageous.

At a unitary price of 99 MAD, CL RDT is not advantageous.

At a unitary price of 100 MAD, CL RDT is not advantageous.

At a unitary price of 101 MAD, CL RDT is not advantageous.

At a unitary price of 102 MAD, CL RDT is not advantageous.

At a unitary price of 103 MAD, CL RDT is not advantageous.

At a unitary price of 104 MAD, CL RDT is not advantageous.

At a unitary price of 105 MAD, CL RDT is not advantageous.

At a unitary price of 106 MAD, CL RDT is not advantageous.

At a unitary price of 107 MAD, CL RDT is not advantageous.

At a unitary price of 108 MAD, CL RDT is not advantageous.

At a unitary price of 109 MAD, CL RDT is not advantageous.

At a unitary price of 110 MAD, CL RDT is not advantageous.

At a unitary price of 111 MAD, CL RDT is not advantageous.

At a unitary price of 112 MAD, CL RDT is not advantageous.

At a unitary price of 113 MAD, CL RDT is not advantageous.

At a unitary price of 114 MAD, CL RDT is not advantageous.

| **import** matplotlib.pyplot **as** plt  *# reminder of the names and value for the parameters*  *# previous_knowledge*  *#1 CL RDT only at the PHC 1*  *#2 CL RDT only at the Lab 1*  *#3 CL RDT + Microscopy confirmation by a lab tech 1*  *#4 CL RDT only at the Lab + driver + patient transfer + 40 Transport 1*  *#5 PPrevious scenario 4 + 20 Administr cost 1 #6 CL RDT done twice at the PHC with 1HP + 62MAD 2nd RDT cost 1*  *# Define different scenarios* scenarios **=** [  (1, 30, 1, 0, 0, 0, 0, 0),  (1, 30, 1, 1, 0, 0, 0, 0),  (1, 75, 2, 1, 0, 0, 40, 0),  (1, 30, 1, 1, 1, 1, 40, 0),  (1, 30, 1, 1, 1, 1, 40, 20), (1, 60, 1, 0, 0, 0, 0, 62)  ]  *# Labels for the scenarios* scenario_labels **=** [  "Scenario 1 CL RDT only at the PHC",  "Scenario 2 CL RDT only at the Lab",  "Scenario 3 CL RDT + Microscopy confirmation by a lab tech",  "Scenario 4 CL RDT only at the Lab + driver + patient transfer + 40 Transport",  "Scenario 5 Previous scenario 4 + 20 Administr cost",  "Scenario 6 CL RDT done twice at the PHC with 1HP + 62MAD 2nd RDT cost" ]  *# Iterate through scenarios* **for** scenario, label **in** zip(scenarios, scenario_labels):  rdt_costs **=** [] previous_knowledge, hp_working_time, hp_needed, main_lab_tech_involved, self_tr **for** rdt_price **in** range(5, 115): *# Assuming a range from 5 to 114*  rdt_cost **=** calculate_rdt_cost(previous_knowledge, hp_working_time, hp_neede rdt_costs**.**append(rdt_cost)  plt**.**plot(range(5, 115), rdt_costs, label**=**label)  plt**.**axhline(y**=**microscopy_cost, color**=**'r', linestyle**=**'--', label**=**"Microscopy Cost 11 plt**.**xlabel("Unitary Price of CL RDT (MAD)") plt**.**ylabel("Overall CL Diagnostic Cost (MAD)") plt**.**legend() plt**.**title("Comparison of Overall CL Diagnostic Cost for Different Scenarios") plt**.**xlabel("Unitary Price of CL RDT (MAD)") plt**.**ylabel("Overall CL Diagnostic Cost (MAD)") plt**.**title("Comparison of Overall CL Diagnostic Cost for Different Scenarios") plt**.**legend(bbox_to_anchor**=**(1.05, 1), loc**=**'upper left') *# Places the legend outside* plt**.**show() |
| --- |

# In [144…


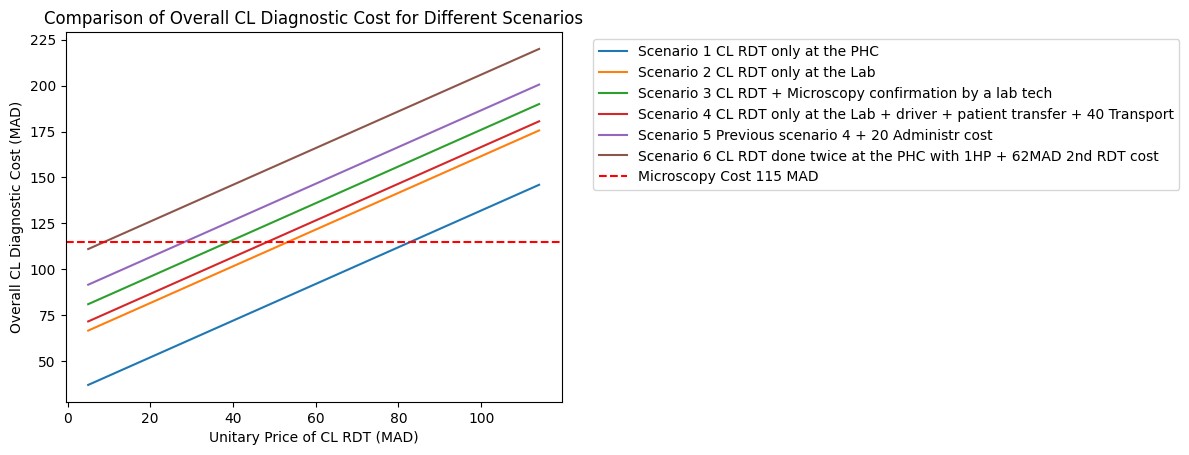


| *# Set the figure size* plt**.**figure(figsize**=**(15, 8))  *# Define a list of grayscale colors*  colors **=** ['k', '0.6', '0.2', '0.4', '0.2', '0.8']  *# Define a list of line styles*  line_styles **=** ['--', '-.', '-', ':', '-.','-']  *# Iterate through scenarios*  **for** idx, (scenario, label, color, linestyle) **in** enumerate(zip(scenarios, scenario_l rdt_costs **=** [] previous_knowledge, hp_working_time, hp_needed, main_lab_tech_involved, self_tr **for** rdt_price **in** range(5, 115): *# Assuming a range from 15 to 114*  rdt_cost **=** calculate_rdt_cost(previous_knowledge, hp_working_time, hp_neede rdt_costs**.**append(rdt_cost)  plt**.**plot(range(5, 115), rdt_costs, color**=**color, linestyle**=**linestyle, label**=**labe  *# Annotate the lines with numbers*  x_position **=** 116 **if** idx **%** 2 **==** 0 **else** 5 y_position **=** rdt_costs[**-**1] **if** idx **%** 2 **==** 0 **else** rdt_costs[0] y_offset **=** **-**5 **if** idx **%** 2 **!=** 0 **else** 0  plt**.**annotate(str(idx), (x_position, y_position **+** y_offset), fontsize**=**12, ha**=**'ri  plt**.**axhline(y**=**microscopy_cost, color**=**'0.5', linestyle**=**'--', label**=**"Microscopy Cost plt**.**xlabel("Unitary Price of CL RDT (MAD)") plt**.**ylabel("Overall CL Diagnostic Cost (MAD)")  plt**.**title("Comparison of Overall CL Diagnostic Cost for Different Scenarios")  *# Create the legend with a smaller font size*  legend **=** plt**.**legend(bbox_to_anchor**=**(1.05, 1), loc**=**'upper left') **for** text **in** legend**.**get_texts(): text**.**set_fontsize(9)  plt**.**tight_layout() plt**.**show() |
| --- |

# In [145…


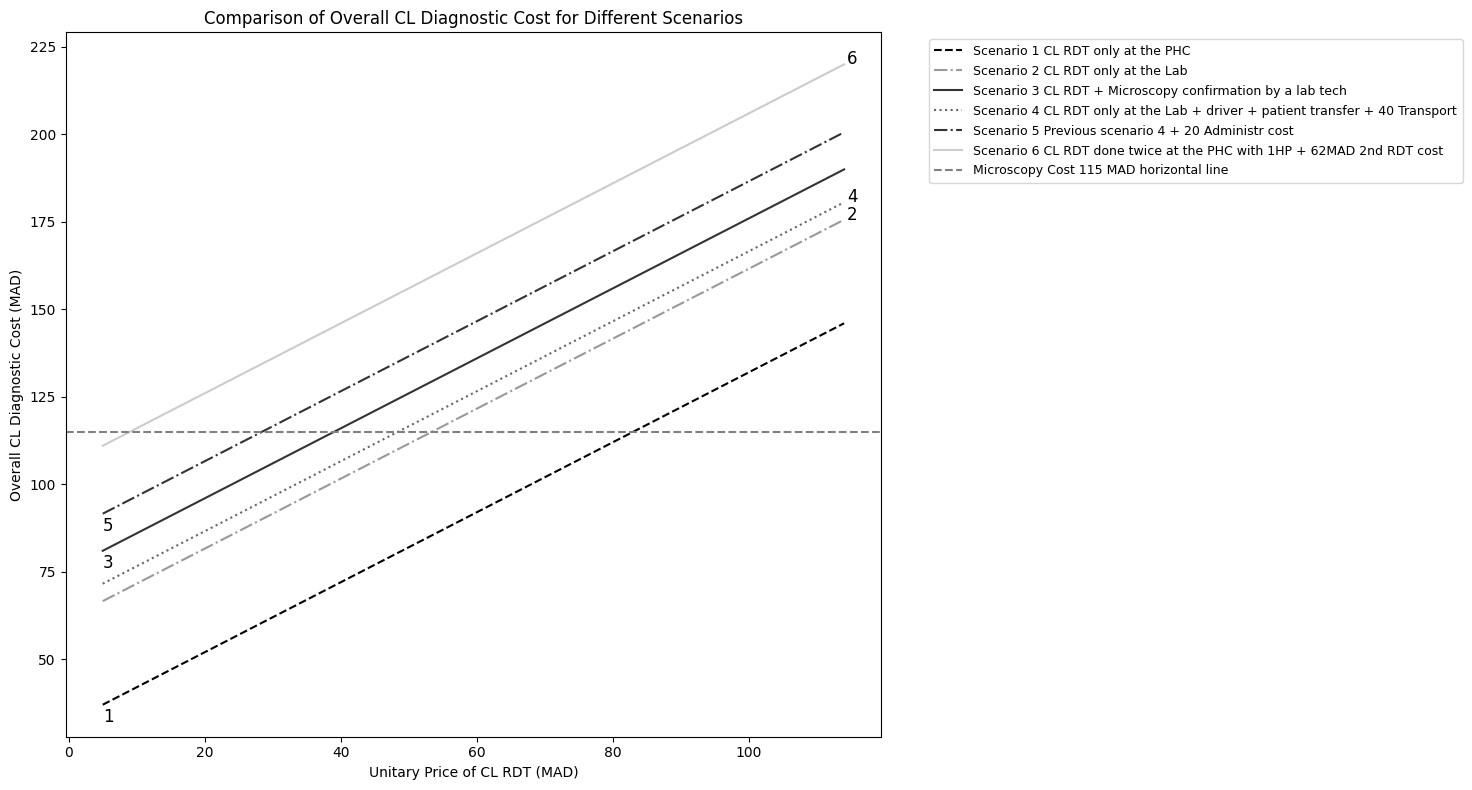


| *# reminder of the names and some parameters*  *# CLRDT Se 68% Sp 94% PPV 95% NPV 64%*  *# Microscopy Se 65% Sp 100% PPV 100% NPV 16%*  *# Benefit value for test accuracy (= True positive rate + true negative rate) TPR=S*  *# prevalence*    *#1 CL RDT only at the PHC 0.5*  *#2 CL RDT only at the Lab 0.5*  *#3 CL RDT + Microscopy confirmation by a lab tech 0.5*  *#4 CL RDT only at the Lab + driver + patient transfer + 40 Transport 0.5*  *#5 Previous scenario 4 + 20 Administr cost 0.5 #6 CL RDT done twice at the PHC with 1HP + 62MAD 2nd RDT cost 0.5*  benefits **=** [  *# Benefit values for scenarios 1 to 6*  0.81 , 1 , 0.9, 1,  0.855, 0.5, 0.7, 1,  0.94 , 0.7, 0.5, 1,  0.855, 0.3, 0.2, 1,  0.855, 0.1, 0.1, 1,  0.855, 0.9, 0.7, 1  ] |
| --- |

# In [146…

| *# Importing necessary libraries* **import** matplotlib.pyplot **as** plt **import** numpy **as** np  *# Function to calculate RDT cost*  **def** calculate_rdt_cost(previous_knowledge, hp_working_time, hp_needed, main_lab_tec diagnostic_tool **=** 1 *# Representing CL RDT*  output **=** (  52.3  **-** 35.8 ***** diagnostic_tool |
| --- |

# In [147…

| **+** 7.4 ***** previous_knowledge  **+** 0.4 ***** hp_working_time   - 3.9 ***** hp_needed   **+** 29.6 ***** main_lab_tech_involved   - 2.4 ***** self_transfer **+** 7.1 ***** driver_transfer   **+** 0.007 ***** patient_transport_cost  **+** 1.0 ***** admin_moh_cost  )  **return** output  *# Scenarios* scenarios **=** [  (1, 30, 1, 0, 0, 0, 0, 0),  (1, 30, 1, 1, 0, 0, 0, 0),  (1, 75, 2, 1, 0, 0, 40, 0),  (1, 30, 1, 1, 0, 0, 40, 20),  (1, 30, 1, 1, 1, 1, 40, 20),  (1, 60, 1, 0, 0, 0, 0, 62)  ]  *# Benefits for each scenario (replace with actual values)* benefits **=** [0.81, 0.855, 0.94, 0.855, 0.855, 0.855]  *# Labels for the scenarios* scenario_labels **=** [  "Scenario 1 CL RDT only at the PHC",  "Scenario 2 CL RDT only at the Lab",  "Scenario 3 CL RDT + Microscopy confirmation by a lab tech",  "Scenario 4 CL RDT only at the Lab + driver + patient transfer + 40 Transport",  "Scenario 5 Previous scenario 4 + 20 Administr cost",  "Scenario 6 CL RDT done twice at the PHC with 1HP + 62MAD 2nd RDT cost" ]  *# Define a list of grayscale colors*  colors **=** ['k', '0.6', '0.2', '0.4', '0.2', '0.8']  *# Define a list of line styles*  line_styles **=** ['--', '-.', '-', ':', '-.','-']  *# Initialize the plot* plt**.**figure(figsize**=**(16, 9))  *# Iterate through scenarios to calculate and store the Cost-Benefit Ratios (CBRs)* reference_cbrs **=** [] *# To store CBRs of the reference scenario (Scenario 3)* **for** scenario, label, color, linestyle, benefit **in** zip(scenarios, scenario_labels, c cbrs **=** [] **for** rdt_price **in** range(5, 115):  rdt_cost **=** calculate_rdt_cost(*****scenario) **+** rdt_price cbr **=** benefit **/** rdt_cost cbrs**.**append(cbr)  *# Store CBRs for the reference scenario (Scenario 3)*  **if** label **==** "Scenario 3 CL RDT + Microscopy confirmation by a lab tech":  reference_cbrs **=** cbrs reference_avg_cbr **=** np**.**mean(cbrs) |
| --- |

| *# Plot the Normalized CBRs*  **for** scenario, label, color, linestyle, benefit **in** zip(scenarios, scenario_labels, c cbrs **=** [] **for** rdt_price **in** range(5, 115):  rdt_cost **=** calculate_rdt_cost(*****scenario) **+** rdt_price cbr **=** (benefit **/** rdt_cost) **/** reference_avg_cbr *# Normalizing CBR*  cbrs**.**append(cbr)  plt**.**plot(range(5, 115), cbrs, color**=**color, linestyle**=**linestyle, label**=**f"{label}  *# Add vertical and horizontal lines for visual reference*  plt**.**axvline(x**=**79, color**=**'g', linestyle**=**'--', label**=**"CL RDT unitary cost at 79 MAD v plt**.**axvline(x**=**54, color**=**'r', linestyle**=**'--', label**=**"CL RDT unitary cost at 54 MAD v Benefit_threshold_scenario3 **=** 1  plt**.**axhline(y**=**Benefit_threshold_scenario3, color**=**'b', linestyle**=**'--', label**=**"Benefi  *# Label axes and add title*  plt**.**xlabel("Unitary Price of CL RDT (MAD)") plt**.**ylabel("Normalized Cost-Benefit Ratio")  plt**.**title("Comparison of Normalized Cost-Benefit Ratios for Different Scenarios")  *# Add legend outside the figure*  plt**.**legend(bbox_to_anchor**=**(1.05, 1), loc**=**'upper left')  *# Show the plot* plt**.**show() |
| --- |


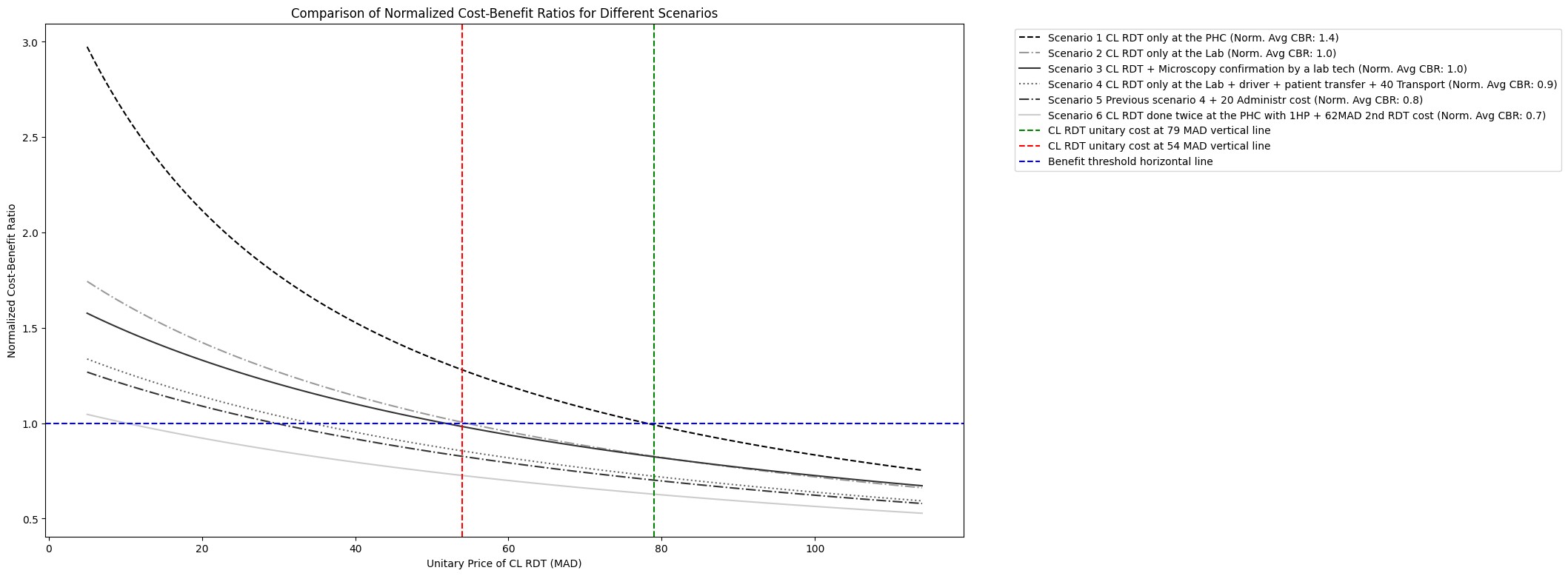

Supplement: S1 Codes — (DOCX) [file pgph.0002534.s012.docx]
