# Supplementary material for: Micro-costing from healthcare professional’s perspective and acceptability of cutaneous leishmaniasis diagnostic tools in Morocco: A mixed-methods study
Source: PLOS Glob Public Health. 2024 Mar 28;4(3):e0002534. doi: 10.1371/journal.pgph.0002534 (PMC10977798; doi:10.1371/journal.pgph.0002534)
Supplement: Attachment — Submitted filename: 1 IBennis&al R1- Response to Reviewers 26 01 2024.docx [file pgph.0002534.s013.docx]

**Reviewer #1:** Congratulations to the authors for their work. Please see below comments for consideration.

>>>> We deeply appreciate the reviewer's valuable feedback and constructive suggestions. We are dedicated to refining our manuscript and are confident that the proposed revisions will significantly elevate its quality, aligning closely with the reviewer's insightful recommendation.

1. Cross-check the entire document for correct grammar and punctuation, e.g." In North Africa, Cutaneous Leishmaniasis" reveals inappropriate use of capital letters

>> Thank you for your constructive feedback and for highlighting the importance of grammatical precision in our manuscript. We used two grammar-checking tools to ensure consistency and accuracy throughout the manuscript. Also, the inapropriate use of capital letters was corrected into the full manuscript.

<<<<<<<<<<<<<<<<<<<<<<<<<<<<<<<<<<<<<<<<<<<<<<<<<<<<<<<<<<<<<<<<<<<
2. Authors are advised to justify their selection of the intervention test.

In the discussion section, it is highlighted that real-time PCR stands out for its superior accuracy across various sampling techniques, notably in the case of scraping smears. This positions it as a potential reference standard for diagnosing cutaneous leishmaniasis (CL). However, the complexity of real-time PCR's operational procedures presents a challenge, particularly when compared to the simplicity of Rapid Diagnostic Tests (RDT). Additionally, other diagnostic methods like Loop-Mediated Isothermal Amplification (LAMP) and nested PCR are currently being refined and developed. Despite the ongoing development of LAMP, its standard operating procedures are complex, posing difficulties for non-laboratory technicians. This complexity is underscored in Taslimi's&al 2023 and Chicharro&al 2023 publications, which detail the LAMP assay AND Nested Real-Time PCR procedures to detect leishmania species. The steps for LAMP include sample collection (specifically skin samples from suspected individuals), DNA extraction, primer design targeting *L. tropica's* kinetoplast DNA, setting up the LAMP reaction mixture, performing the isothermal amplification at a consistent temperature (around 65°C), visual detection of the reaction through colourimetric readout or gel bands, and result interpretation based on these observations.

Then, the alternative is the RDT that was found with same accuracy compared to Microscopy in Moroccan context, despite being less sensitive than Microscopy in other countries with differrents leishmania species.

<<<<<<<<<<<<<<<<<<<<<<<<<<<<<<<<<<<<<<<<<<<<<<<<<<<<<<<<<<<<<<<<<<<

3.I recommend statements that are not author's own should be supported by references. The paragraph below for example hardly has references
" In Eastern Mediterranean countries where Cutaneous Leishmaniasis is endemic, the standard
laboratory test involves light microscopy examination of a smear prepared by scraping a
suspected lesion. This method enables the visual identification of parasites following Giemsa
stain colouration. Ideally, results are obtained in approximately one and a half hours when
colouration and microscopy readings are performed directly. However, in many remote areas
where people rely on primary health centres (PHCs), patients often require two to three PHCs
and lab visits over several days to receive microscopy results."

>>> thank you for this suggestion. Now please find the same paragraph with the suitable added references.

In Eastern Mediterranean countries where Cutaneous Leishmaniasis is endemic, the standard laboratory test involves light microscopy examination of a smear prepared by scraping a suspected lesion. This method enables the visual identification of parasites following Giemsa stain colouration (7). Ideally, results are obtained in approximately one and a half hours when colouration and microscopy readings are performed directly (8)…….

7 Saki J, Amraee D, Ghasemian M. Comparison of Nested-PCR and Loop-Mediated Isothermal Amplification for Cutaneous Leishmaniasis Diagnosis. Jundishapur J Microbiol [Internet]. 2019 [cited 2024 Jan 10];12(8). Available from: https://brieflands.com/articles/jjm-94171.

8 De Vries HJC, Schallig HD. Cutaneous Leishmaniasis: A 2022 Updated Narrative Review into Diagnosis and Management Developments. Am J Clin Dermatol. 2022 Nov 1;23(6):823–40.

<<<<<<<<<<<<<<<<<<<<<<<<<<<<<<<<<<<<<<<<<<<<<<<<<<<<<<<<<<<<<<<<<<<

4.What is the burden of leishmaniasis in the selected areas of study?

>>> This information is provided as supplementary material for the number of cases and reinforced by a new sup_inf 6bis showing the overall number of CL in Morocco

**Sup_inf 6. Cutaneous leishmaniasis 2019's epidemiological data in the study area**

Between the 3^rd^ and the 4^th^ paragraph, the following sentence is added:

in Morocco, where CL are endemic with each five years cyclicity for ZCL and almost a sustainable yearly trend of ACL cases (Sup_inf 6 & 6bis), people rely for CL diagnostics on primary health centres (PHCs). Then, patients often require two to three PHCs and lab visits over several days to receive microscopy results.

<<<<<<<<<<<<<<<<<<<<<<<<<<<<<<<<<<<<<<<<<<<<<<<<<<<<<<<<<<<<<<<<<<<

5.It is not clear why the sampling process led to a predominantly high number of nurses? Are they the ones who mainly offer clinical care to the patients? Perhaps a brief overview of health service proivision in Morocco will help put the findings in context

>>> the CL treatment is provided by nurses . the physicians examine the patient and prescribe the diagnostic and or the treatment needed.

Both the diagnostic and the treatment of CL are the specific duties of nurses.

Then, the rapid diagnostic test was mainly done by nurses, also when the scraping of CL lesions are done in primary healh centers only nurses do it.

<<<<<<<<<<<<<<<<<<<<<<<<<<<<<<<<<<<<<<<<<<<<<<<<<<<<<<<<<<<<<<<<<<<

6.How exactly were the interviews conducted? Did all the 40 participate in an interview all at once? How was consenting done and confidentiality maintained?

>>>> Thank you for this question, effectively all the 40 health professionals participated one by one in indepth individual audio recordred interviews

we added sentences (highlighted in yellow) and reorganised the data collection and sampling strategy subsection to clarify more this information.

Data collection and sampling strategy

Between 26 August 2019 and 28 January 2020, healthcare professionals (HPs) who frequently managed CL patients at each visited primary health centre and district laboratory were invited for face-to-face audio-recorded individual in-depth interviews (46 HPs in total) (Sup_Inf 2).

The principal investigators' team included two male doctors and two male administrative nurses experienced in participating in previous scientific studies. Each investigator worked in one of the four selected provinces. They received a full-time, one-week training workshop on qualitative interviews, cost-effectiveness, and an overview of the project objectives, rationale, research questions, and data collection tools. Each investigator respected the confidentiality criteria, and ethical consent was obtained before starting the video demonstration and the interview record. ~~These discussions were led by a team of four principal investigators.~~ Structured face-to-face interviews were conducted using a topic guide organised by thematic questions (Sup_Inf 3). Then, initial interview discussions involved 40 HPs, some with prior experience using the CL-RDT, some knowledgeable about it, and others unaware of its existence. All participants watched a video-recorded demonstration explaining the use of the CL-RDT (as shown in Sup_Inf 1) before answering the main interview questions.

>>>>Additionally, the document's section on ethical considerations and good publication practices expressly mentions that: 'All HPs provided formal written consent to participate in the study'

<<<<<<<<<<<<<<<<<<<<<<<<<<<<<<<<<<<<<<<<<<<<<<<<<<<<<<<<<<<<<<<<<<<

7.I propose supporting quotes be added to the text to support the deductions made from the qualitative arm of the research and summary table of the qualitative findings highlighting the themes and sub-themes be added to the text

>>> As recommanded by the reviewer, we added fiftheen supporting quotes into the main text (from page 16 to page 20).

In addition we added a summary table of the qualitative finding highlighting the main finding of themes and subthemes added at the qualitative results subsection as shown at the begining of the subsection in page 16 :

**The qualitative results linked to CL diagnostic.**

Initial interviews were conducted by the principal investigator's team, followed by six focused interviews at the end. No differences in responses based on gender were found. However, differences were observed in other attributes, as explained below and summarised in table 7.

**Table 7: Summary of the health professional's in-depth interviews' analysis of cutaneous leishmaniasis diagnostics**

| Main Theme | Sub-Theme | Description |
| --- | --- | --- |
| Diagnostic process and efficiency | **Workload comparison** | The workload for CL-RDT is lower than for microscopy.  Healthcare professionals prefer CL-RDT to improve diagnostics and increase patient compliance.  Concerns about increased workload in rural PHCs due to healthcare professional shortages. |
|  | **Usefulness in different areas** | The perceived usefulness of CL-RDT varies: it is more appreciated in *L. tropica* areas (Tinghir, Sefrou provinces) compared to *L. major* areas (Errachidia, Ouarzazate). |
|  | **Training and familiarity with CL-RDT** | Laboratory technicians find the procedure straightforward, but not doctors and nurses.  Familiarity with CL-RDT among healthcare professionals leads to ease and proficiency in use. |
|  | **Practicality of CL-RDTs** | Mixed opinions on the practicality of CL-RDTs in primary health centres.  Some find it easy and beneficial, reducing transportation costs for patients.  Others rely on clinical decisions and epidemiological trends.  Training and consistent testing across health centres and laboratories are suggested. |
|  | **Comparative analysis with microscopy** | CL-RDT does not require formal training and can be performed by any healthcare professional, potentially testing more patients per day than microscopy.  Microscopy requires at least two weeks of training and can be emotionally stressful. |
| Expected patients' preferences | **Acceptability and comfort** | Acceptability of CL-RDT varies based on healthcare professionals' familiarity with it.  Concerns about discomfort from the dental broach are alleviated by local anaesthetic.  No reported pain or damage from dental broach use. |
|  | **Waiting time for results** | The acceptable waiting time for microscopy results is 5 hours, but the actual wait can be 2 to 4 working days, sometimes over 15 days.  Delays are due to technical issues, lack of training, and scheduled weekly activity organisation.  Patients are unwilling to wait for microscopy results, preferring quicker CL-RDT results. |
| Economic aspects and healthcare policy considerations | **Resource allocation and training** | Insufficient trained healthcare professionals or laboratory technicians could result in service interruptions.  Suggestions for improved motivation mechanisms from the Ministry of Health and training one healthcare professional and one lab technician in each endemic area. |
|  | **Use in remote areas and by non-health workers.** | Non-health workers performing RDTs in remote areas are not accepted due to concerns about storage conditions, ambient temperature, expiration dates, and regulations.  Pharmacists and private drugstores could conduct CL-RDTs, provided tests are reimbursed by the health coverage system. |
|  | **Patient's willingness to pay** | Patients prefer undergoing the diagnostic process at the same PHC to avoid extra costs like transportation.  The suggested cost for CL-RDTs in drugstores should not exceed 50 MAD.  Patients consent to treatment when positive test results indicate increased compliance with CL-RDT. |

<<<<<<<<<<<<<<<<<<<<<<<<<<<<<<<<<<<<<<<<<<<<<<<<<<<<<<<<<<<<<<<<<<<8.I propose that in the discussion section, the authors compare the similarities and differences in their studies with other similar studies and conclude by giving their thoughts on new directions in advocacy, research and clinical practice

>>> Thank you for the valuable, as there are not yet of micro-costing studies targeting CL Diagnostic tools, we added the newest articles published during this three last months with introducing as suggested by the reviewer a paragraphs to discus similarities and differences, and proposd further researchs directions.

All the four main added paragraphs in the discussion section are highlighted in yellow :

………………. The overall CL diagnostic costs are impacted by the strategies defined for CL control (which could demand targeting the best diagnostic confirmation scenarios) and the general acceptability of the diagnostic tool by healthcare professionals (which can influence how often the tool is used or manipulated).

Our findings align with very few studies that targeted CL diagnostic costs rather than determining only the test's accuracy. Indeed, the studies from Peru and Sri Lanka agreed on the need for low-cost CL diagnostic tests, defined (as 4–5 USD per test (12, 25).

However, our study extends these cost insights by quantifying the micro-costing advantage of CL-RDT and exploring its acceptability in the Moroccan context. Unlike another study from Columbia, which focused on the cost analysis of PCR kDNA test for mucocutaneous leishmaniasis diagnostic, that could be recommended for future research for localised cutaneous leishmaniasis areas (26).

Our study highlighted at least the same costs of CL RDT compared to Microscopy. This is almost the same finding in a previous study where RDT was comparably cost-effective to Microscopy at reference clinics (53 USD) but offered ease of use, making it cost-effective at peripheral levels (48 USD) with no differences in costs between the RDT and Microscopy (22).

………………………. The currently available CL Detect Rapid Test fails to meet the requisite diagnostic performance standards of reaching 95% sensitivity for point-of-care waiting for the next generation of CL-RDT based on antigen detection that should differentiate between *L.tropica, L.major*, and other species (1,29,30).

……………………… The psychosocial burden prevalent in some areas, where there is an urgent need to minimise scarring through prompt treatment, underscores the importance of using diagnostic tools like the current CL Detect Rapid Test (CL RDT). With its high specificity, as indicated by the review's pooled analysis showing an overall specificity of 94% [87-97%], the CL RDT or similar rapid diagnostic tools can provide quick answers, essential for ruling out CL (30). This is especially relevant in Morocco, reflecting a pattern observed in Sri Lanka, where, for the *L. donovani* species, the RDT demonstrated a sensitivity of 36% but a specificity of 100% (25). A recent study from Sri Lanka highlights the significant public health concern posed by the psychosocial impact of leishmaniasis, necessitating a holistic approach that addresses the physical, psychological, and social aspects of affected CL patients (39).

………………………………….. It is imperative to recognise the intricate cost structure categorised into fixed, variable, and combined costs as conceptualised in our context (**Figure 1**), each playing a pivotal role in the overall financial framework. Notably, the green indicators in our analysis emphasise the dynamic nature of these costs, influenced by factors such as healthcare professionals' acceptability of diagnostic tools, policy-driven CL control strategies, and the specific diagnostic confirmation scenarios employed. The general acceptability of diagnostic tools among healthcare professionals directly affects their usage frequency, thereby influencing variable costs. Strategically defined control policies can optimise the diagnostic workflow, resulting in more efficient use of resources and reduced costs. Furthermore, the selection of diagnostic confirmation scenarios determines the allocation of both fixed and variable resources, underpinning the importance of strategic decision-making in the economic management of CL diagnostics.

These findings advocate for policy shifts towards adopting CL-RDT in endemic areas in the Moroccan context. Future research should focus on expanding the accessibility of CL-RDT and exploring its application in diverse epidemiological settings. Depending on the CL species and clinical presentation, the cost-effectiveness of CL-RDT should be compared to microscopy for multiple lesions.

Secondly, both microscopy and CL-RDT should improve their sampling procedures to obtain higher parasite loads with fewer invasive, repetitive lesions, increasing confidence in both tests. The sensitivity of CL-RDT was low for *L.ethiopica* in Ethiopia and *L.donovani* in Sri Lanka (25,44). The sampling site of lesions could influence parasitological diagnosis sensitivity (45). Therefore, the development of improved testing methods is crucial.

<<<<<<<<<<<<<<<<<<<<<<<<<<<<<<<<<<<<<<<<<<<<<<<<<<<<<<<<<<<<<<<<<<<

Finally, we have formatted the references in accordance with the Vancouver style to align with the PLoS Journal's criteria.Thereby, the new four references added :

7.	Saki J, Amraee D, Ghasemian M. Comparison of Nested-PCR and Loop-Mediated Isothermal Amplification for Cutaneous Leishmaniasis Diagnosis. Jundishapur J Microbiol. 2019;12(8).

8.	de Vries HJC, Schallig HD. Cutaneous Leishmaniasis: A 2022 Updated Narrative Review into Diagnosis and Management Developments. Am J Clin Dermatol. 2022 Nov;23(6):823–40.

26.	Castillo-Rodríguez L, Ovalle-Bracho C, Díaz-Jiménez D, Sánchez-Vanegas G, Muvdi-Arenas S, Castañeda-Orjuela C. Cost-effectiveness analysis of Mucosal Leishmaniasis diagnosis with PCR-based vs parasitological tests in Colombia. PLoS One. 2019 Nov 4;14(11):e0224351.

30.	Gebremeskele BT, Adane G, Adem M, Tajebe F. Diagnostic performance of CL Detect rapid-immunochromatographic test for cutaneous leishmaniasis: a systematic review and meta-analysis. Systematic Reviews. 2023 Dec 20;12(1):240.

Additionally, two references was removed from the manuscript due to firstly discrding the sentence referend and secondly due to an inactive link, which necessitated its replacement.

~~[37]	I. N. Schuermeyer, E. Sieke, L. Dickstein, T. Falcone, and K. Franco, 'Patients with challenging behaviors: Communication strategies',~~ *~~CCJM~~*~~, vol. 84, no. 7, pp. 535–542, Jul. 2017, doi: 10.3949/ccjm.84a.15130.~~

Either for the first one replaced due to inactive link

~~[1] WHO, 'Target product profile for a point of care diagnostic test for dermal Leishmaniasis', 2022. https://apps.who.int/iris/rest/bitstreams/1422309 (accessed 16 August 2023).~~

<<<<<<<<<<<<<<<<<<<<<<<<<<<<<<<<<<<<<<<<<<<<<<<<<<<<<<<<<<<<<<<<<<<

<<<<<<<<<<<<<<<<<<<<<<<<<<<<<<<<<<<<<<<<<<<<<<<<<<<<<<<<<<<<<<<<<<<

<<<<<<<<<<<<<<<<<<<<<<<<<<<<<<<<<<<<<<<<<<<<<<<<<<<<<<<<<<<<<<<<<<<

**Reviewer #2:** Thank you for the opportunity to review this manuscript. The manuscript should be re-organised to achieve publication quality. Please find my comments and suggestions below:

>>>> We express our sincere gratitude for the insightful comments and suggestions offered by the reviewer. We hope the valuable contribution have significantly improved both the readability and coherence of our manuscript in its current form.

* Provide more details motivating the study, i.e. what is the CL prevalence, distribution of disease (age, sex, etc.), what are the estimated coverage rates for CL diagnostic tests, what is the national management protocol for CL

>>> Dear Reviewer, thank you for your inquiry regarding the estimated coverage rates for Cutaneous Leishmaniasis (CL) diagnostic tests within the Moroccan healthcare system. We would like to provide some insights into how the system is structured and operates, particularly concerning the diagnosis of CL.

The Moroccan healthcare system is based on a pyramidal structure featuring an extensive network of public primary healthcare facilities. This network is designed to provide equitable coverage across the entire geographical territory, encompassing both urban and rural areas. It is at these primary healthcare facilities that the initial contact with patients suspected of having lesions indicative of Cutaneous Leishmaniasis typically occurs.

In terms of diagnostic testing for CL, it is important to note that currently, the primary method employed for diagnostic confirmation is microscopic testing. The use of rapid diagnostic tests (RDTs) for CL is indeed feasible at this level of care, yet their implementation is not done at present. The primary healthcare facilities serve as crucial points for both the identification of suspected CL cases and the subsequent diagnostic processes.

We have included in Supplementary Information 6 and 6 bis detailed data on the specific distribution of cutaneous leishmaniasis types, including variations by age, sex, and year. This addition aims to provide a more comprehensive understanding of the epidemiological aspects of CL in Morocco.

And we added in the background section (page 3) the following small paragraph:

The Moroccan healthcare system for CL management employs a pyramidal network of primary healthcare facilities, ensuring equal access in urban and rural areas. Initial examinations of suspected CL cases predominantly use microscopic testing for diagnosis, a widely implemented and fundamental method. Although the potential of CL rapid diagnostic tests (RDTs) could be acknowledged, their integration into the system is still pending, making microscopy the primary diagnostic tool in use (9).

<<<<<<<<<<<<<<<<<<<<<<<<<<<<<<<<<<<<<<<<<<<<<<<<<<<<<<<<<<<<<<<<<<<

* Methods: it should be clearly stated that the analysis unit is the HP practice (n=46)

>>>> We added this sentence in page 5: The analysis unit in this study is the healthcae professionals. At the end of the Population and Study Settings Method subsection.

In page 5 we also added: ….at each visited primary health centre and district laboratory were invited for face-to-face audio-recorded individual in-depth interviews (46 HPs in total) (Sup_Inf 2).

In page 6 we added: The principal investigators' team revisited the 40 initial HPs to check the self-administered quantitative questionnaire.

<<<<<<<<<<<<<<<<<<<<<<<<<<<<<<<<<<<<<<<<<<<<<<<<<<<<<<<<<<<<<<<<<<<
* Remove unnecessary details (order of authors when describing responsibilities, credentials of individuals capturing data in Excel)
>>> Removed as recommanded by the reviewer.

<<<<<<<<<<<<<<<<<<<<<<<<<<<<<<<<<<<<<<<<<<<<<<<<<<<<<<<<<<<<<<<<<<<

* The results section needs reorganisation:

>>>Indeed the qualitative part was reorganised depending mainly on the reviewer 1 comments.

>>> In addtion, the quantitative section is now reogranised to satisfy the recommandations of the reviewer 2 as follow :
* The results section should begin by enumerating the cost categories that emerged from the qualitative analysis, including their meaning as it might not be clear to everyone (e.g. what is transport cost?)

>>>> we added five subsections for all the quantitaitve results section : Costs categoristion , Average of CL lesion number on the cost analysis of CL-RDT and microscopy , Comparative analysis of time, workload, and transport costs in CL diagnostics: CL-RDT vs. Microscopy , Regression analysis of CL diagnostic costs , Sensitivity assessment of CL diagnostic costs across different scenarios (More explanation is below)

<<<<<<<<<<<<<<<<<<<<<<<<<<<<<<<<<<<<<<<<<<<<<<<<<<<<<<<<<<<<<<<<<<<

* Cost results should be grouped into standard categories, namely "fixed" and "variable". For example, workload is a variable cost.

>>> We added a new figure 1 to descripe the costs categories used in this study, with an explanation of all the cateogrisation components, as shown :

The green part of the figure includes:

- Healthcare Professional (HP) General Acceptability of the Diagnostic Tool: This element suggests that the overall usage and cost-effectiveness of a diagnostic tool for CL are heavily influenced by how well healthcare professionals accept it. If the tool is widely accepted, it will likely be used more frequently, thus affecting the variable costs associated with diagnostics.
- Policy CL Control Strategy Defined: This signifies the strategic decisions made at a policy level which affect the overall costs of CL diagnostics. A well-defined control strategy can streamline the diagnostic process, potentially leading to more efficient resource allocation and reduced overall costs.
- Diagnostic Confirmation Scenarios: These are specific cases or circumstances under which the diagnosis of CL is confirmed. The scenarios chosen for confirmation can impact the fixed and variable costs due to the different resources and time required for each scenario.

**Figure 1: Cost categorisation flowchart for CL diagnostic tools from a healthcare professional's perspective**

>>> In addition We added the explanation of those costs into the method section page 6 and 7

In the pursuit of standardisation and transparency in micro-costing for cutaneous leishmaniasis (CL) diagnosis from a healthcare professional's perspective, our study delineates costs into fixed, variable, and combined categories (17).

Fixed costs refer to expenses that do not change with the activity level within a specific range. In this study, fixed costs include:

- Equipment and laboratory material costs: These are the expenditures for acquiring, maintaining, and using equipment and materials necessary for CL diagnosis in laboratories.
- Salaries of healthcare professionals: Regular wages for medical staff, including laboratory technicians, physicians, and nurses, independent of the volume of CL tests conducted.
- HP working time: The financial equivalent of healthcare professionals' time devoted to CL diagnosis, which includes their fixed salaries.
- Distance costs to reach facilities: These cover the travel-related expenses incurred when patients or samples must be transported to diagnostic centres.
- Driver transfer: The cost of transport services to move patients or samples to and from the healthcare facilities.
- Patient self-transfer: Out-of-pocket expenses for patients travelling to healthcare facilities for diagnosis.
- Administrative MoH costs: Operational costs handled by the Ministry of Health for the administrative side of CL diagnostics, including data handling and record-keeping. In our framework, these administrative costs are not charged to the patient.

Variable costs change with the level of activity. Key variable costs in this study are:

- Time testing: The duration-dependent cost associated with performing the CL diagnostic test.
- Diagnostic strip or slide unit purchased: The expense of individual diagnostic strips for RDT or microscopy slides is essential for the CL testing process.
- Additional transport costs: Extra travel costs related to the diagnostic procedure, potentially necessitated by additional testing or the inadequacy of initial diagnostic tools.

Combined costs: Integrating fixed and variable costs is pivotal in comprehensive micro-costing analysis (18).

- Transportation costs: An aggregation of all travel expenses, encompassing distances to facilities and additional costs, which fluctuate based on the diagnostic tool used.
- Workload diagnostic costs: Costs impacted by the complexity of tool manipulation and the time investment by healthcare staff in the diagnostic process.
- Diagnostic confirmation scenarios represent the potential additional costs incurred when further testing is needed to confirm CL diagnoses, influencing the total costs.
- Overall CL diagnostic costs: This is the cumulative impact of the cost categories from the initial patient interaction to the final diagnosis confirmation.

The cost categorisation flowchart for CL diagnostic tools from a Healthcare professional's perspective is presented in **Figure 1**.

<<<<<<<<<<<<<<<<<<<<<<<<<<<<<<<<<<<<<<<<<<<<<<<<<<<<<<<<<<<<<<<<<<<

* In general, the results section seems disorganised. Results seem to appear out of nowhere and therefore it is hard to read. In addition to describing cost categories, the quantitative section should begin by outlining how results are presented.

>>>> In accordance with the reviewer's suggestion, we have restructured the results section to incorporate distinct subsections. The initial subsection now introduces a matrix categorizing costs, delineating specific expenditures along with their respective ranges. This addition is designed to enhance the transparency and comprehension of our cost analysis. The corresponding paragraph and table have been included to provide a detailed breakdown of these financial aspects :

Costs categorisation

As shown in **Table** 2, which includes the main results of the cost catégorisation values comparing the microscopy to the CL-RDT, the cost analysis for diagnosing cutaneous leishmaniasis in Morocco using microscopy and CL-RDT, while initially calculated under the assumption of one lesion per patient, needs adjustment considering the actual lesion count. Both methods start with identical equipment, and asepsis costs 10 MAD for a single lesion. Additional consumables for CL-RDT bring its specific costs to about 3 MAD more. Microscopy slides are cheaper at 5 MAD compared to 62 MAD for a CL-RDT strip. The overall unitary cost is lower for microscopy at 15 MAD than 75 MAD for CL-RDT. However, CL-RDT shows a significant cost advantage in workload, averaging 27 MAD, much lower than microscopy's 99 MAD. This extends to overall diagnostic costs (100 MAD for CL-RDT vs. 115 MAD for Microscopy) and transport costs, which are lower for CL-RDT. Administrative costs from the Ministry of Health are assumed to be zero for both methods.

**Table 2: Detailed micro-costing of microscopy and rapid diagnostic test for cutaneous leishmaniasis from a healthcare professional's perspective**

| **Costs Categories** | **Cost value/Range for Microscopy** | **Cost value/Range for CL-RDT** | **Notes** |
| --- | --- | --- | --- |
| One lesion sample equipment protection and asepsis cost | 10 MAD [4,6-22] | 10 MAD [4,6-22] | Necessary consumables for diagnosing one lesion are similar to CL microscopy and CL-RDT. Detailed descriptions are available in Supporting Information 7* |
| Additional dental broach, reaction cup and pipette tip and tube for CL-RDT cost |  | 3 MAD [1,6-7] | The study defines consumables within the year of study; for specifics, refer to Supporting Information 7* |
| Microscopy unit slide cost | 5 MAD [2-9] |  | The study defines consumables within the year of study; for specifics, refer to Supporting Information 7* |
| CL-RDT unit strip cost |  | 62 MAD [46-102] | The study defines consumables within the year of study; for specifics, refer to Supporting Information 7* |
| Microscopy unitary cost | 15 MAD [7-31] |  | Cost calculations include the addition of the necessary consumables as outlined, with lines 1 and 3 being pertinent to different diagnostic tools. |
| CL-RDT unitary cost |  | 75 MAD [52-131] | Cost calculations include the addition of the necessary consumables as outlined, with lines 1, 2, and 4 pertinent to different diagnostic tools. |
| Workload costs | 99 MAD [42-276] | 27 MAD [7-67] | Costs are partly based on the healthcare professionals' time required to perform the diagnostic process without accounting for transfer or transport delays, noting that microscopy is typically more expensive and time-consuming than CL-RDT (as detailed in Table 4). |
| Overall Diagnostic Cost | 115 MAD [58-292] | 100 MAD [79-131]** | The aggregated costs combine fixed and variable elements, including the workload and unitary equipment costs, and vary according to the unitary cost of the RDT strip (refer to Table 4 for scenario-specific costs). |
| Average transport cost | 122 MAD [8-360] | 67 MAD [6-190] | Geographical distance-related expenses and additional transport costs depend on various assumptions and scenarios, fully described in Table 5. |
| Maximal transport cost | 224 MAD [8-720] | 124 MAD [8-380] | Geographical distance-related expenses and additional transport costs depend on various assumptions and scenarios, fully described in Table 5. |
| Administrative MoH cost | 0 | 0 | Administrative costs, as determined by the Ministry of Health regulations, are assumed to be free of charge in this context. |

(*) Supporting Information 7 (**Sup_Inf 7**) lists the additional equipment and laboratory materials needed for microscopy and RDT CL diagnostics.

(**) if the unitary strip cost is 62 MAD (1 USD = 9,6 MAD)

>>>> Acknowledging the reviewer's input, the second results subsection has been appropriately renamed to reflect the updated content more accurately.

Average of CL lesion number on the cost analysis of CL-RDT and microscopy

The cost analysis of CL-RDT and microscopy is appreciably affected by the mean number of CL lesions per patient, with a noted range from 1.7 in Tinghir to 2.2 in Errachidia, Ouarzazate, and Sefrou, as indicated in **Sup_Inf 6**. Such variability implies a potential escalation in actual costs from the base figures, predominantly for CL-RDT. Theoretically, considering the average lesion count, the per-patient cost for CL-RDT might increase commensurately, influencing its cost-effectiveness relative to microscopy. For example, in localities with a 2.2 lesion average, the diagnostic expenses for CL-RDT could surpass double the projected cost for a singular lesion. Nonetheless, practical clinical approaches, as qualitatively described, involve selecting the most representative lesion for sampling and reserving additional lesion sampling for cases with initial negative results.

>>>> Then, we have now expanded the results section with a newly titled subsection for enhanced clarity and depth.

Comparative analysis of time, workload, and transport costs in CL diagnostics: CL-RDT vs. microscopy

This subsection present the same paragrphs explaining table 3 and table 4.

With a new addtion of table 5	to explain results finding of transport	costs. As mentionned below .

>>>> We added also a small paragraph to explain this new table 5.

**Table 5** reports that patients using CL-RDT typically required fewer healthcare visits, with most needing only a single visit, a significant reduction from the average of 2.3 visits for microscopy. This trend extends to maximum visit counts, transport costs, and additional transport expenses, where CL-RDT consistently demonstrates lower averages and ranges. While the differences in some transport costs are not statistically significant, the overall pattern favours CL-RDT.

**Table 5: Descriptive analysis of transport costs for CL-RDT and Microscopy**

|  | **Diagnostic tool manipulated** | | | | **N*** | | **Mean** | | **SD** | | **Min** | | **Max** | | **p-value**** | |
| --- | --- | --- | --- | --- | --- | --- | --- | --- | --- | --- | --- | --- | --- | --- | --- | --- |
| Minimal Needed visits to PHC and/or Lab to get CL diagnostic | |  | Microscopy |  | 34 |  | 2.3 |  | 0.4 |  | 2 |  | 3 |  |  |  |
|  | |  | CL Detect Rapid Test ™ |  | 6 |  | 1.0 |  | 0.0 |  | 1 |  | 1 |  | <.0001 |  |
| Maximal Needed visits to PHC and/or Lab to get CL diagnostic. | |  | Microscopy |  | 34 |  | 3.4 |  | 0.5 |  | 2 |  | 4 |  |  |  |
|  | |  | CL Detect Rapid Test ™ |  | 6 |  | 2.3 |  | 0.5 |  | 2 |  | 3 |  | 0.0008 |  |
| One-time transport cost from PHC to Lab (Assumption of 4 MAD as a minimum for walking without any public transport use) | |  | Microscopy |  | 34 |  | 58.6 |  | 55.4 |  | 4 |  | 180 |  |  |  |
|  | |  | CL Detect Rapid Test ™ |  | 6 |  | 58.6 |  | 73.8 |  | 4 |  | 190 |  | 0 .8 |  |
| Additional minimal transport cost (assumption PHC is near to each home with no mandatory need to go to Lab) | |  | Microscopy |  | 34 |  | 20.8 |  | 30.4 |  | 0 |  | 80 |  |  |  |
|  | |  | CL Detect Rapid Test ™ |  | 6 |  | 10.3 |  | 15.4 |  | 0 |  | 40 |  | 0.7 |  |
| Additional maximal transport cost (assumption PHC is near to each patient home with the mandatory need to go to Lab) in MAD | |  | Microscopy |  | 34 |  | 224.0 |  | 227.6 |  | 8 |  | 720 |  |  |  |
|  | |  | CL Detect Rapid Test ™ |  | 6 |  | 124.6 |  | 145.9 |  | 8 |  | 380 |  | 0.3 |  |
| Average transport cost in MAD | |  | Microscopy |  | 34 |  | 122.4 |  | 108.7 |  | 8 |  | 360 |  |  |  |
|  | |  | CL Detect Rapid Test ™ |  | 6 |  | 67.5 |  | 71.6 |  | 6 |  | 190 |  | 0.1 |  |
| (*) It indicates that among the 40 healthcare professionals participating in the study, 6 had prior hands-on experience with the CL-RDT. These experienced individuals were from six distinct healthcare facilities, as elaborated in Supporting Information 2. Conversely, the remaining 34 participants were exclusively acquainted with the CL-RDT procedure through a video demonstration.  (**) The p-value was calculated by ANOVA unidirectional for non-parametric tests due to not respecting normality and homoscedasticity conditions. (1 USD = 9,6 MAD)  SD= standard deviation, Min = Minimum, Max= Maximum | | | | | | | | | | | | | | |  |  |

>>>> The final two subsections of the results, which address the specifics of our cost analysis and its implications, will be detailed in the forthcoming responses to the reviewer's comments.

Regression analysis of CL diagnostic costs:

Sensitivity assessment of CL diagnostic costs across different scenarios

<<<<<<<<<<<<<<<<<<<<<<<<<<<<<<<<<<<<<<<<<<<<<<<<<<<<<<<<<<<<<<<<<<<

* Considering rephrasing this sentence because it is not clear: "The overall number of CL lesions needing diagnosis is 1.7 to 2.2 times the total number of reported CL persons."

>>>> As mentionned now in the results section, this sentence was rephrased as :

Average of CL lesion number on the cost analysis of CL-RDT and microscopy

The cost analysis of CL-RDT and microscopy is appreciably affected by the mean number of CL lesions per patient, with a noted range from 1.7 in Tinghir to 2.2 in Errachidia, Ouarzazate, and Sefrou, as indicated in **Sup_Inf 6**. Such variability implies a potential escalation in actual costs from the base figures, predominantly for CL-RDT. Theoretically, considering the average lesion count, the per-patient cost for CL-RDT might increase commensurately, influencing its cost-effectiveness relative to microscopy. For example, in localities with a 2.2 lesion average, the diagnostic expenses for CL-RDT could surpass double the projected cost for a singular lesion. Nonetheless, practical clinical approaches, as qualitatively described, involve selecting the most representative lesion for sampling and reserving additional lesion sampling for cases with initial negative results.

~~Supplementary Information 6 (~~**~~Sup_Inf 6~~**~~) contains CL epidemiological data from the same~~

~~lesions. The overall number of CL lesions needing diagnosis is 1.7 to 2.2 times the total number of reported CL persons.~~

<<<<<<<<<<<<<<<<<<<<<<<<<<<<<<<<<<<<<<<<<<<<<<<<<<<<<<<<<<<<<<<<<<<

* It should be clearly stated what the numbers inside brackets mean. Range? Minimum and maximum?
>>> Done (Value/range) appears now in table 2 and (range) appears in table 4 and 5. The abrevaiation Min and Max are notified as minimum and maximum.

<<<<<<<<<<<<<<<<<<<<<<<<<<<<<<<<<<<<<<<<<<<<<<<<<<<<<<<<<<<<<<<<<<<

* This phrase is wrong: " The p-values represent the healthcare professionals' (HP) general acceptability of the two diagnostic tools, calculated using a unidirectional ANOVA with Fisher's classic test." That is not what a p-value is. Please explain or restate your affirmation.

>>>> We reformulate this sentence as :

~~The p-values represent the healthcare professionals' (HP) general acceptability of the two diagnostic tools, calculated using a unidirectional ANOVA with Fisher's classic test.~~ The p-values obtained from a unidirectional ANOVA with Fisher's classic test indicate the statistical significance of the differences in costs associated with the diagnostic tools, which can influence healthcare professionals' (HP) preferences or acceptability of these tools.

<<<<<<<<<<<<<<<<<<<<<<<<<<<<<<<<<<<<<<<<<<<<<<<<<<<<<<<<<<<<<<<<<<<

* The regression analysis needs more justification, including an explanation of the variables included in the right-hand side of the equation.

>>>> Thank you for the reviewer valuable feedback regarding our regression analysis in Table 6 (previousely table 4). We appreciate the opportunity to clarify the justification for the **Table 6** provides a detailed linear regression model, demonstrating the factors influencing the overall CL cost, including workloads, unitary equipment and laboratory material costs.

The linear regression model presented in Table 6 was designed to comprehensively analyse the factors influencing the overall cost of CL diagnostics. Each predictor variable was selected based on its relevance to the cost dynamics of CL diagnosis:

- Diagnostic tool manipulated: This variable captures the cost differential between using microscopy and the CL Detect Rapid Test™. It is significant in our model, indicating that choosing diagnostic tools substantially impacts cost.
- Previous Knowledge of CL-RDT: Addresses whether prior familiarity with CL-RDT influences the diagnostic cost. Though not statistically significant, it has been included for its potential practical relevance.
- HP working time: Reflects the impact of healthcare professionals' time on the cost, a crucial aspect given the differing time requirements of the diagnostic methods.
- HP needed for the diagnostic test: Explores whether needing more than one healthcare professional affects the cost.
- Main laboratory tech involved: Assesses the cost implications of involving a primary laboratory technician in the diagnostic process.
- Patient motif of transfer for diagnosis process: Includes two scenarios - sampling in the PHC without patient transfer Vs MoH driver involvement - to examine transportation's role in overall costs.
- Average transport cost: Considered to understand how transport expenses contribute to the total diagnostic cost.
- Administrative MoH cost: Although minimal, it has included to ensure a comprehensive cost model.

<<<<<<<<<<<<<<<<<<<<<<<<<<<<<<<<<<<<<<<<<<<<<<<<<<<<<<<<<<<<<<<<<<<

* It is not surprising that the R-squared of the linear regression model is so high, given that the model is decomposing total cost into its constituent parts. Therefore, the high R-squared is an artifact of the data. Contribution of individual cost components is influenced by a) the share of each cost category, and b) how much variation was reported by HP.

>>>> Thank you for this observation regarding the high R-squared value of our linear regression model. We understand the concern that the high R-squared might appear as an artifact given the model's design, which essentially decomposes the total cost into its constituent parts. However, we wish to clarify the purpose and the analytical approach of our model to demonstrate its validity and relevance.

The primary objective of our model is not just to decompose the total cost but to understand the relative influence and significance of each cost component in the context of CL diagnostics. This understanding is crucial for healthcare decision-making, particularly in optimising resource allocation and identifying key cost drivers. In addition, our model was designed to capture the complexity of cost components in CL diagnostics. Each predictor in the model represents a distinct aspect of the diagnostic process, and their combined effect explains a significant portion of the variability in the total cost.

We have conducted additional validation check, including sensitivity analysis to ensure the robustness of our findings. These checks confirm that the model's predictions are reliable.

<<<<<<<<<<<<<<<<<<<<<<<<<<<<<<<<<<<<<<<<<<<<<<<<<<<<<<<<<<<<<<<<<<<

* The scenarios are poorly explained and seem to appear out of nowhere in the results section. It is not enough to reference Figure 2, please explain what those scenarios mean in the body of the text.

>>>> We added a new subsection as follow :

Sensitivity assessment of CL diagnostic costs across different scenarios

We conducted a sensitivity analysis to assess the robustness of our cost findings rigorously. This analysis not only underscores the reliability of our results but also sheds light on the relative impact of various factors under different conditions.

The scenarios were meticulously constructed to reflect a range of practical situations in CL diagnostics, providing a comprehensive view of potential cost variations influenced by different clinical and logistical factors.

The results from these scenarios, informed by our sensitivity analysis, illustrate the dynamic nature of CL diagnostic costs, highlighting key variables that exert the most significant influence. The key parameters were:

- Previous knowledge of CL-RDT (binary variable [0, 1]): This parameter considers whether healthcare professionals have prior experience or knowledge of using CL-RDT.
- Diagnostic tool used (Microscopy = 1, CL-RDT = 0, binary variable): Differentiates the costs based on whether microscopy or CL-RDT is used for diagnosis.
- Health professional working time ([20, 62] minutes): Represents the time healthcare professionals spend performing the diagnostic process.
- Number of Health professionals needed (Just one =1, More than one =2, binary variable): Assesses the impact of requiring more than one professional for the diagnostic test.
- Patient transport modalities:
  - Self-transfer (Sampling in PHC without patient transfer = 1, binary variable): When patients visit the PHC themselves without needing additional transportation.
  - Driver transfer (MoH Driver involved = 1, binary variable): When transportation involves a driver, possibly increasing costs.
- Patient transport cost ([14 MAD, 432 MAD]): The financial burden of patient transportation.
- Involvement of main laboratory technician (binary variable [0, 1]): Examines the cost effect of involving the main laboratory technician in the diagnostic process.
- Administrative MoH cost ([0 MAD, 1 MAD]): As regulated by the Ministry of Health, administrative costs are minimal in our context.

**Figure 2** displays the total sensitivity indices for crucial parameters influencing the overall CL diagnostic cost at two distinct unitary costs of CL-RDT: 45 MAD and 74 MAD, corresponding to the overall CL-RDT cost of 83 MAD and 112 MAD, respectively. The analysis highlights that the 'Diagnostic Tool Manipulated' parameter substantially impacts 49% to 76% of the overall CL diagnostic cost. Conversely, the 'Main Laboratory Tech Involved' parameter plays a significant but varying role, contributing 34% to 17% of the overall CL diagnostic cost. Other parameters, including 'HP Working Time,' exhibit more modest influences. At the same time, factors like 'Previous Knowledge of CL-RDT,' 'HP Needed,' 'Self Transfer,' 'Driver Transfer,' 'Patient Transport Cost,' and 'Administrative MoH Cost' contribute marginally.

Python analysis defined the CL-RDT unitary cost range compared to microscopy cost (115 MAD). As explained in the method section, we employed the SALIb library with the Sobol method to quantitatively assess the sensitivity of our cost model to changes in the various input parameters, ensuring a thorough and robust analysis (**Sup_Inf 11**).

Then, real-world scenarios were simulated to examine how parameter changes could affect the cost of diagnosing cutaneous leishmaniasis, thereby calculating cost-benefit rates.

<<<<<<<<<<<<<<<<<<<<<<<<<<<<<<<<<<<<<<<<<<<<<<<<<<<<<<<<<<<<<<<<<<<

* Qualitative results: really good. Uncommon in costing literature and it really adds value to the paper.

>>>> Thank you for the reviewer comment

<<<<<<<<<<<<<<<<<<<<<<<<<<<<<<<<<<<<<<<<<<<<<<<<<<<<<<<<<<<<<<<<<<<

* Conclusion section should summarise the cost analysis, i.e. average cost for RDT and Microscopy.

Our study indicates that Rapid Diagnostic Tests (CL-RDT) have a micro-costing advantage over microscopy in diagnosing cutaneous leishmaniasis. The average cost of CL-RDT is 100 MAD (ranging from 79 to 131 MAD), compared to microscopy, which averages 115 MAD (ranging from 58 to 292 MAD). This cost includes workload and transport expenses, where CL-RDT demonstrates significantly lower costs, particularly in transport.

Moreover, healthcare professionals prefer CL-RDT due to its ease of use and quick results. In rural areas endemic to CL, the time efficiency and micro-costing advantage of CL-RDT make it a precious tool for CL control management. The most significant micro-costing benefit is observed when CL-RDT is used as the primary healthcare centre's primary diagnostic method, especially if the RDT strip cost remains below 79 MAD.

In anticipation of forthcoming versions with improved sensitivity, deploying the next-generation CL-RDT in resource-limited settings is advocated.

>>>> Either the last sentnce of the conclusion of the Abstract section was modified :

In conclusion, the forthcoming CL-RDT, expected to feature enhanced sensitivity, is advocated for deployment ~~is recommended as an alternative diagnostic tool~~ in resource-limited endemic areas.

<<<<<<<<<<<<<<<<<<<<<<<<<<<<<<<<<<<<<<<<<<<<<<<<<<<<<<<<<<<<<<<<<<<

* Table 2: should read "interval" rather than "interval", and "min" instead of "inf", "max" instead of "sup"

>>>> The new number of the previous table 2 is now table 3. That is corrected as asked by the reviewer.

<<<<<<<<<<<<<<<<<<<<<<<<<<<<<<<<<<<<<<<<<<<<<<<<<<<<<<<<<<<<<<<<<<<

* Table 3: needs better footnotes, i.e. what does SD mean? not clear for everyone that it stands for standard deviation. Also, not clear what the sample sizes represent e.g. 34 vs 6?

>>>> The new number of the previous table 3 is now table 4. SD abreviation is explained as standard deviation, the Min as minimum and the Max as maximum.

For the sample size we clarified in the method section (in page 6) that the unit cost dats was calculated from the answers of 40 HP, one by each of the 40 primary health care centres and district labs involved.

In our study, the micro-costing included the direct enumeration and costing of all inputs consumed for patient diagnostic and program implementation. . These costs were then correlated with unit cost data from the 40 primary healthcare facilities, including centres and district laboratories, where the study's health professionals worked (19)

Then, six participants from the six healthcare facilities (namely Sekoura, Chahid Baha, Tarmight, Ferkla, Ait Ouassif and Kelaa Megouna B) used and manipulated effectively the RDT while the other participants 34 didn’t do it as shown in Sup Inf 2.

In addition, we have appended a footnote to Table 4 to clarify the sample size details. (*) It indicates that among the 40 healthcare professionals participating in the study, six had prior hands-on experience with the CL-RDT. These experienced individuals were from six distinct healthcare facilities, as elaborated in Supporting Information 2. Conversely, the remaining 34 participants were exclusively acquainted with the CL-RDT procedure through a video demonstration.

<<<<<<<<<<<<<<<<<<<<<<<<<<<<<<<<<<<<<<<<<<<<<<<<<<<<<<<<<<<<<<<<<<<

* Table 4:
* Do authors mean "standard error" instead of "standard deviation"?
* In a multivariate regression model, the intercept is not the base cost. Also, the footnote doesn't match the table (48.2 MAD vs 52.3)
* R-squared is not a measure of model robustness.

>>>> table 4 in previous version become now table 6.

Thank you for the reviewer observation. Pointing out that the term 'standard error' is appropriate in this context, and not 'standard deviation'. The table indeed refers to the 'standard error' of the estimated coefficients, which measures the accuracy with which the coefficients are estimated in the regression model. Table 6 corrected.

Thank you for highlighting the interpretation of the intercept in a multivariate regression model. The intercept represents the expected value of the dependent variable when all predictors are at their reference levels, not necessarily the base cost. Our model's intercept should indeed be interpreted in the context of the specific variables and their relation to the overall cost.

Then we made the following change:

~~Intercept baseline (*): The constant term of the model, or the base cost, is 48.2 MAD (Moroccan Dirham).~~

Intercept*: The intercept of the regression model, representing the expected value of the overall CL diagnostic cost when all predictor variables are at their reference levels, is 52.3 MAD (Moroccan Dirham).

>>>> For the last reviewer comment ( * R-squared is not a measure of model robustness. )

Thank you for this insightful comment regarding the interpretation of R-squared. We acknowledge that R-squared is not a measure of model robustness but rather indicates the proportion of variance in the dependent variable explained by the independent variables in our regression model.

Then, the initial sentence in the footnote table 6 was corrected by the following one :

The model's explanatory power is highlighted by its high coefficient of determination (R² = 0.98), which indicates that the independent variables in the model account for 98% of the variability in the overall CL cost.

<<<<<<<<<<<<<<<<<<<<<<<<<<<<<<<<<<<<<<<<<<<<<<<<<<<<<<<<<<<<<<<<<<<

Thank you again for all the reviewers insightful comments, which have undoubtedly strengthened our manuscript.
